# Presence Is Reality: Rethinking Virtual and Real-World Consciousness

**DOI:** 10.1177/17456916251414030

**Published:** 2026-02-10

**Authors:** Oliver Singleton, Aikaterini Fotopoulou

**Affiliations:** Clinical, Educational and Health Psychology Research Department, Division of Psychology and Language Sciences, University College London

**Keywords:** presence, virtual reality, VR, predictive processing

## Abstract

The sense of presence is typically defined as the feeling of “being there” in a virtual environment, whereas the sense of reality is defined as the ability to discriminate between real and unreal phenomena. We challenge this rigid dichotomy, arguing that presence and reality can be considered conceptually, mechanistically, and phenomenologically continuous. We first demonstrate that both cognitive sciences and virtual reality (VR) studies use the terms inconsistently and interchangeably. We then go on to identify and combine perceptual and cognitivist accounts of presence, arguing that presence, like reality, is likely to be formed from integrative mechanisms. We then go further to identify converging psychophysical findings from the two fields in multisensory integration, self-embodiment, and agency. This is further supported by results from preliminary neuroimaging studies, indicating a shared frontolimbic substrate for generating the feeling of “realness.” This reconceptualization has significant implications, including validating the use of VR as a tool for studying the sense of reality and its clinical disorders. We conclude by advocating for directly comparing these phenomena in future research to systematically test for their functional and neural equivalence.

The sense of presence is broadly referred to as the feeling of existing within one’s surrounding environment, overwhelmingly in the context of virtual reality (VR) research (Metzinger, 2003; Sanchez-Vives & Slater, 2005). VR can be defined in a multitude of ways, but we regard a definition of VR to be the one generated by Burdea and Coiffet (2024)—a simulation in which computer graphics and other display modalities are used to create a synthetic world that responds to user inputs (Burdea & Coiffet, 2024). Although this definition of VR may seem restrictive because it excludes other systems that may also be considered early examples of VR technology (such as the Sensorama from 1962; Jones & Dawkins, 2018), we use it here because we are primarily interested in discussing presence in virtual worlds—a phenomenon that specifically takes place within a virtual environment generated using a VR headset and other display modalities such as sound, touch, and olfaction.

Similarly, the sense of reality is broadly referred to as the ability to determine whether perceptual phenomena originate internally from the mind or externally from the environment (Johnson & Raye, 1981) or, put more simply, the ability to discriminate an unreal experience from a real experience (Fortier, 2018). This ability is often used as a central criterion in the assessment of psychiatric health (Drori et al., 2020; Metzinger, 2014; Ratcliffe, 2008). However, although a full review of definitions is outside the scope of this article, it is important to note that individual definitions used by different researchers in both the sense of presence and the sense of reality vary wildly and often overlap in functionality, already blurring the line between these two concepts.

In this article, we propose that presence and reality are continuous—mechanisms that generate one sense are the same mechanisms that generate the other sense, and disruptions of each sense cause identical phenomenology. Consequently, research that claims to investigate the sense of presence investigates the same phenomenon as studies exploring the sense of reality, and vice versa. We demonstrate this by first showing how researchers themselves do not seem to apply these terms with much logical consistency. We then argue for a unitary and integrative account of presence incorporating both lower order perceptual and higher order cognitive mechanisms. Such integrative mechanisms seem to mirror the integrative mechanisms of the senses of reality, further blurring any distinction between the two. We then go on to discuss similarities in the aims and findings of studies investigating the mechanisms of these two concepts and highlight cases of converging evidence. Moreover, we outline preliminary neuroscientific studies that suggest that there is also a significant overlap in brain mechanisms between these two senses. Last, we conclude that VR studies that investigate the sense of presence could theoretically be used to effectively investigate concepts relating to the sense of reality, including depersonalization-derealization disorder (DPDR) and exposure therapies. We also outline the broader implications such a conclusion has on philosophy and the cognitive sciences.

## One or Two Concepts?

One immediate suggestion that the sense of presence and the sense of reality are more similar than their definitions imply is the fact that researchers themselves often do not know which term to use. We argue here that many researchers seem to instead arbitrarily pick a term on the basis of their overall field of study (VR research or cognitive-science research), and even then this is not consistent across labs within each field. Furthermore, review articles and introductions to each topic commonly incorporate literature that uses the other term, rendering any distinction between presence and reality practically meaningless.

The sense of reality has a long history in philosophy and other fields and far too many definitions and constructions to list here (see Fortier, 2018). However, there have been attempts to synthesize this literature, focusing on some of its more fundamental properties, such as the basic premise that there is something that it is like for an object, a physical state, or an environment to feel real as opposed to unreal (Fortier, 2018). It is worth noting, however, that such attempts also make no distinction between the sense of presence and the sense of reality, citing multiple VR studies as evidence for different constructions of the sense of reality.

Definitions and constructions of presence are just as varied, but there have been fewer attempts to synthesize this literature. One widely accepted definition of presence in the field of VR research is as the sense that one feels present within a virtual world (Berkman & Akan, 2019; International Society for Telepresence Research, 2025; Sanchez-Vives & Slater, 2005). Nevertheless, the term “presence” in cognitive science can describe either the feeling of being fully present and receptive within a current moment (Deshmukh, 2022) or, more commonly, the subjective sense that one exists within one’s surrounding environment (Seth et al., 2012).

We are therefore faced with two terms that appear distinct on the surface: a sense of reality concerning the authenticity of the external world and a sense of presence concerning the existence of the self within that world. However, what this difference means both phenomenologically and in practice is unclear. As a result, it seems that many researchers have taken to interchangeably using the term “presence” in the cognitive-science sense or the term commonly used in VR research, sometimes substituting one or either of these uses with the term “reality” without explanation (Drori et al., 2020; Felton & Jackson, 2022; Fortier, 2018; Seth, 2014; Seth et al., 2012; Suzuki et al., 2023).

**Fig. 1.**
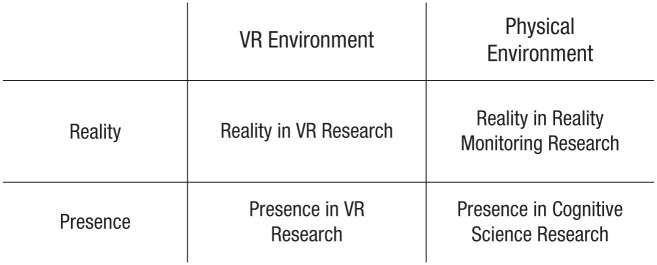
Different ways in which “reality” and “presence” are used interchangeably across both VR research and research in the cognitive sciences. VR = virtual reality.

This means that interdisciplinary work that uses but does not clarify such terms could mean either one of four things, or any of their combinations (Fig. 1): “reality” in terms of VR research, “reality” in terms of cognitive science, “presence” in terms of cognitive science, or “presence” in terms of VR research. Although this conceptual uncertainty may stem from an actual overlap in the phenomena under consideration, to our knowledge no study has systematically examined this possibility. As a result, some authors treat the concepts “presence” and “reality” as separate, whereas others refer to them interchangeably, and hence any interdisciplinary synthesis of the literature faces obvious difficulties. Accordingly, we aim to systematically examine the relation between these terms across fields, focusing on their use, mechanisms, and phenomenology.

## Identifying and Combining Perceptual and Cognitive Accounts of Presence

As mentioned previously, the sense of reality and the sense of presence are typically considered distinct. Part of the reason for this distinction is that they rely on different mechanisms that are specialized according to whether the experienced environment is physical (reality) or virtual (presence). There are two dominant views of presence that attempt to explain why this distinction matters: the perceptual account and the cognitivist account. In this section, we argue that neither account in isolation supports a coherent view of presence and that the various findings of VR research can be explained only by an integrative account of presence that combines bottom-up perceptual processes with top-down cognitive modulation. Such a view of presence situates it within the same predictive, hierarchical architecture that underlies the sense of reality—suggesting that both experiences are generated by the same integrative mechanisms (which we go on investigate in the following section). In doing so, this challenges the assumption that presence is unique to virtual environments, framing it instead as just one instance of the broader process by which the brain constructs the feeling of “realness” in other environments.

It may be reasonable to assume that the technology and characteristics of a VR environment, as opposed to a physical environment, create unique conditions that give rise to the sense of presence that is somehow distinct from the sense of reality. There are two broad theoretical accounts of presence in VR that may explain what may be unique about VR environments compared with real environments—the perceptual account and the cognitivist account. The perceptual account of presence in VR focuses on the importance of accurate and realistic sensorimotor information and its impact on presence (Sanchez-Vives & Slater, 2005; Slater, 2018), more commonly referred to as presence at the “perceptual” level. The cognitive account of presence in VR focuses on presence as being primarily constructed through higher order mental processes (Nunez & Blake, 2001; Sas & O’Hare, 2003), more commonly referred to as presence at the “cognitive level.” For simplicity, we refer to the “cognitive level” when we mean higher order mental processes and the “perceptual level” when we mean base perception (raw, unprocessed sensory input) because brain sciences typically characterize both higher order mental processes and base perception as facets of cognition.

Theorists of the cognitive stance argue that individual human factors such as empathy, creative imagination, willingness to suspend disbelief, and absorption alongside technical factors such as image quality and visual angle (for a full list of factors, see Sas & O’Hare, 2003) are the primary drivers for experiencing presence in VR. Such theorists do not entirely ignore the role of base perception (e.g., multisensory congruency, visual fidelity) in the creation of presence in VR but do place a greater importance on higher order cognitive factors (Sas & O’Hare, 2003). However, a primarily cognitivist account of presence does not appear fully supported by the state of current presence literature.

Arguments for the cognitivist stance on presence tend to focus on positive correlations between reported presence levels in VR (as measured by questionnaires) and other traits such as suggestibility (Jicol et al., 2023) and mental imagery (Iachini et al., 2019). Suggestibility and mental imagery, although influenced by low-level perceptual processing, are traits that are often considered to depend on higher order functions such as attentional control, executive function, and working memory (Karpinski & Scullin, 2009; Palmiero et al., 2019).

It has been argued that the prevalence of such correlations is an indicator that these functions are likely to be involved in the creation of presence (Iachini et al., 2019; Jicol et al., 2023) and that presence in VR varies depending on individual cognitive traits (Sas & O’Hare, 2003). These individual cognitive traits can be classified as higher order functions because their recruitment depends on complex mental processes such as theory of mind (empathy; Derntl & Regenbogen, 2014), divergent thinking (creative imagination; Tateo, 2016), cognitive flexibility (which improves the ability to temporarily adapt to alternative realities in the suspension of disbelief), and metacognition (which allows for the regulation of immersion in the suspension of disbelief; Preiss, 2022). However, the direction of causality between these facets of cognition and presence is unknown, meaning this argument for the cognitivist position on presence rests on an association between questionnaire measures of presence and questionnaire measures of greater suggestibility and mental imagery (Iachini et al., 2019; Jicol et al., 2023) that could reasonably be explained by a reverse causality (an immersive virtual experience may incline participants to report greater suggestibility or mental imagery).

By contrast, authors of several other VR studies have argued for the perceptual stance on presence. Indeed, the importance of the role of perceptual properties has been the major focus of presence research for the past 3 decades, with many previous studies supporting the idea that differences in the availability and quality of perceptual information in VR by far have the greatest impact on the sense of presence (for review, see Barranco Merino et al., 2023). However, despite this supposed importance, a vast majority of these studies have focused on vision-only research, with few studies researching nonvisual senses such as olfaction, thermoception, taste, and touch in isolation, leaving many gaps in the current literature (Cornelio et al., 2021; Gallace et al., 2012).

3D audio, for example, is a type of nonvisual VR in which sound contains a spatial quality, instantiating a sense of presence in participants without the need for a visual component (Bormann, 2005; Rumsey, 2021). Nevertheless, such studies have focused mostly on the role of vision combined with audition, finding that congruent multisensory experiences have a large and replicable impact on presence in VR (Cooper et al., 2018; Gallace et al., 2012; Marucci et al., 2021), whereas incongruent multisensory experiences in VR are liable to induce derealization-like qualities (Valzolgher et al., 2018). Similar findings have been found regarding audiovisual stimuli in VR when using standard two-speaker stereo setups, although this type of audio by itself does not normally contain a spatial quality (Cooper et al., 2018).

It is important to note here that when such studies talk of multisensory congruency they do not mean that the presented stimuli were congruent across all possible modalities but rather that they were congruent across the particular modalities of interest presented to participants (e.g., touch and vision or vision and audition). Although this does not directly mirror a typical experience in physical reality in which one is free to interact with an object in any combination of ways, this absence of expected stimuli does not prevent multisensory integration from occurring. For example, the absence of audition in a visuohaptic experiment will not prevent multisensory integration from taking place between the congruent stimuli that are actually present (Cooper et al., 2018; Gallace et al., 2012; Marucci et al., 2021; Peter et al., 2019).

Researchers have also explored the role of touch on presence, although to a far lesser extent and with more mixed results, broadly finding that the addition of touch alongside vision significantly improves reported presence levels (Biocca et al., 2002; Gibbs et al., 2022; Kreimeier et al., 2019). Multiple neurological studies utilizing EEG measures have identified distinct event-related potentials related to perception that positively correlate with individual presence scores (Grassini et al., 2021; Kober et al., 2012; Safikhani et al., 2024).

Last, supporters of the perceptual account of presence have argued that prior knowledge of the virtual nature of their environments is not sufficient to counter the perceptual experience that virtual events are “real.” Multiple studies have examined this phenomenon, identifying significant increases in heart rate and skin conductivity when participants are placed in vertigo-inducing VR simulations compared with nonthreatening virtual environments (Meehan et al., 2002) or VR exposure therapy (VRET) for percepts that also scare them in real life (Bohil et al., 2011). Slater (2018) emphasized the importance of these types of response as indicators of low-level presence, arguing that

it [presence] is a perceptual but not a cognitive illusion, where the perceptual system, for example, identifies a threat (the precipice) and the brain-body system automatically and rapidly reacts (this is the safe thing to do), while the cognitive system relatively slowly catches up and concludes “But I know that this isn’t real.” But by then it is too late, the reactions have already occurred. (p. 432)

These accounts and studies propose that presence is primarily driven by the perceptual rather than the cognitive level. However, it is equally unlikely to be the case that presence is entirely driven at the perceptual level. Individuals do not, for example, typically experience frightening or traumatic events in VR in the same way as in physical environments. Although simulations of traumatic events in VR can indeed elicit similar bodily reactions to traumatic events in reality (Mühlberger et al., 2007), there is a lack of empirical evidence to suggest that such situations can lead to long-lasting mental-health problems such as posttraumatic stress disorder (PTSD), although there are exceptions to this in the form of case reports and clinical VRET studies that we discuss in the Potential Limitations section. This suggests that although VR may elicit bodily responses to virtual simulations that are similar to responses elicited by real-life events, events in VR do not leave the same lasting impact on the user’s mind as if they had occurred in reality. This indicates some level of top-down cognitive modulation of perception in VR environments.

Such top-down modulation, however, is clearly not sufficient to negate the affective impact of virtual environments. This can be seen, for example, in studies that show VR as a promising treatment for PTSD through exposure therapy (Difede & Cukor, 2007; Kothgassner et al., 2019). We therefore conclude here that, although virtual experiences must be modulated by higher order cognitive processes at some point, they may be real enough at some perceptual level to impact participant’s beliefs about the real world or their own past experiences. As we go on to show in the next section, this means that mechanisms for presence are far more likely to be integrative—combining bottom-up perception with top-down cognitive modulation—and therefore further blurring the distinction between modern understandings of presence and reality.

## Integrative Accounts of Presence and Reality

If presence in VR is integrative, then this may suggest that the sense of presence is constructed mechanistically in the same way as the sense of reality. This would be because previous studies and theories in the sense of reality have shown that our experience of the real world is not a direct reflection of objective reality but a partly reconstructed perception shaped by a combination of bottom-up sensory input and top-down higher cognitive modulation (Clark, 2013; Friston et al., 2015; Johnson & Raye, 1981). Although VR environments may sometimes lead to contradictions between perceptual and cognitive levels (e.g., “I know this is virtual, but I feel like it’s real”), this is perhaps no different from how certain dreams, illusions, or hallucinations create experientially real experiences while the perceiver maintains the knowledge of their unreal nature. In this sense, we argue that presence in VR may be characterized as the existing mechanisms of the sense of reality applied to a virtual setting, suggesting that rigid conceptual distinctions between “presence” in VR and “presence” or “reality” in the cognitive sciences are hard to uphold.

It should be noted that although the current article is the first to systematically explore the similarities between the notions of “presence” and “reality” in VR and “presence” and “reality” in the cognitive sciences and suggest that they share some common mechanisms, it is not the first to propose an integrative theory of presence that could be applied to all such cases. Integrative theories of presence that consolidate bottom-up sensorimotor information with top-down higher order modulation have been formulated separately in both the cognitive sciences and VR research.

For example, Seth et al. (2012) proposed such a model for presence in the field of cognitive science in which they outlined a predictive processing account of conscious presence. Predictive processing is a wider influential model in brain sciences that frames mental processes in terms of hierarchical predictions that are sent downstream (top-down processing) and compared against incoming perceptual information from the world (bottom-up processing; Clark, 2013, 2015; Seth, 2014). This comparison then generates prediction errors—mismatches between predictions about the state of the world and incoming perceptual information from the world. These prediction errors prompt the brain to adjust internal models of the environment, minimizing future prediction errors and creating a more accurate internal model of the world.

In Seth et al.’s (2012) predictive processing account of presence, presence is viewed as an affective feeling state that is influenced by a combination of interoception (the perception of the physiological condition of the body; Garfinkel et al., 2013; Khalsa et al., 2018) and exteroception (the perception of the environment via exteroceptive sensory modalities such as vision; audition; taste, which has interoceptive as well as exteroceptive components; and olfaction; Toussaint et al., 2024). According to this model, signals from the sensorimotor system and autonomic system (low-level perceptual information) are separately matched to predictions regarding the state of each system (higher order cognition), with the magnitude of prediction errors arising from differences between the expected and predicted sensorimotor and autonomic states influencing presence. In this model, large prediction errors result in lower presence, whereas smaller prediction errors result in greater presence (Seth et al., 2012).

An alternative integrative model of presence in VR was proposed by Berthiaume et al. (2018) that, although bearing striking similarity to the model proposed by Seth et al. (2012), makes no such reference to models of presence developed in the cognitive sciences. This integrative model of presence in VR places great emphasis on the role of incoming low-level perceptual information—what Seth et al. (2012) would classify as “exteroceptive input”—and how accurately these incoming perceptual signals match expected multisensory congruency (a prediction of the exteroceptive state). The brain then explicitly processes the resulting prediction error to determine whether incoming sensorimotor information is “believable” (a higher order cognitive state formed from a comparison between expectations of sensorimotor properties of low-level perceptual information and actual sensorimotor information and influenced by other higher order cognitive factors such as the ability to suspend disbelief); the resulting prediction error then informs how plausible the VR environment is and therefore the magnitude of the sense of presence within that environment. Again, greater prediction error here reduces presence, whereas a reduced prediction error increases presence (Berthiaume et al., 2018).

Although the integrative model by Berthiaume et al. (2018) differs from the model by Seth et al. (2012) in that it seems to ignore the role of interoception in presence, the exteroceptive pathways, namely the process of the formation of prediction errors from mismatches between expected sensorimotor information and actual incoming sensorimotor information and their influence on the sense of presence, are identical. Although this overlap of theoretical innovation may not be surprising, considering that many such integrative models have been applied to other facets of self-consciousness (Schwengerer, 2019), perception of the external world (Walsh et al., 2020), and other higher order, metacognitive aspects of cognition (Fernández Velasco & Loev, 2024), it is interesting to see no reference made to such a closely parallel field.

Another theoretical model that takes predictive processing as foundational to its explanation of presence is the model developed by Pianzola et al. (2021) that posits that presence is felt when subjects are able to “correctly and intuitively enact (i.e., without the involvement of reasoning) their implicit (predictive processing) and explicit (intentions) embodied predictions” (para. 5). This model builds on work done by Seth et al. (2012) regarding how the brain incorporates interoceptive information in an embodied, predictive processing model. However, the authors argued that the relationship between presence and agency in Seth et al., (2012) model is incorrect and that the reverse is the case. Seth et al. (2012) described agency as being hierarchically at a higher level than presence, meaning that prediction errors concerning presence impact the sense of agency, whereas prediction errors relating to agency do not impact the perception of the sense of presence.

Pianzola et al. (2021) argued that this means that, theoretically, only agency can constrain predictions regarding presence, but in practice predictions related to presence can be enacted even in the case in which prediction errors relate to agency. The authors’ model instead describes presence as a neuropsychological phenomenon in which the goal of presence is to generate agency in the embodied self. This agency then goes on to supervise predictions related to both exteroception and interoception, meaning an agent feels present when they can enact their embodied predictions. Presence, therefore, is characterized here as a general embodied cognitive state. Importantly, this model does not constrain itself to either attempting to describe just cognitive presence, virtual presence, or the sense of reality but positions itself in such a way that it can describe all such instances of presence/reality regardless of whether the environment is mediated or not.

Antti Revonsuo’s seminal book *Inner Presence* (Revonsuo, 2006) is foundational to this modern conceptualization of presence as a construct arising from exteroceptive and interoceptive prediction error. In this work, Revonsuo introduced what he termed the “world simulation metaphor” for conscious presence. He argued that the sense of presence instantiated in dreams, VR, and the real world are identical. When we dream, we do not have the conscious experience of being trapped inside one’s brain—even though this is invariably where the dream takes place. Instead, the environment of the dream is virtually externalized, so the perceiver feels as though the dream takes place within a seemingly real perceptual world in which they have a fully convincing experience of being present within. Revonsuo further argued that the same is true of environments rendered to participants who are wearing head-mounted VR devices. Although the environment the user is in is rendered within the headset, the user nevertheless experiences the environment as if it were an externalized place in which they are physically situated. This type of false externalization fits well within a predictive processing framework in that it posits conscious presence as something biologically evolved by the brain to allow us to simulate our external realities, even when our realities are not external at all.

Coelho et al. (2006) shed more light on this argument by laying out the potential distinguishing features between the type of inner presence described by Revonsuo (2006) and what “rationalist” researchers would describe as “media presence”—a psychological state in which a participant fails to understand the role of technology in their immediate conscious experience in VR (Coelho et al., 2006). Although the authors did not appear to explicitly support one view of presence over the other, they did lay out an argument by Steuer (1992), who contended that, because presence is so foundational to VR to be seen as part of its definition, VR itself should not be defined solely in terms of hardware. We can further expand on this argument by saying that because presence, in Steuer’s terms, is the result of the interaction between the user and the virtual environment, not between the user and the VR technology (by definition, “media presence” stipulates that it can occur only when the user is *unaware* of the technology mediating their experience), then there ceases to be a reason why presence in VR must necessarily be separate from presence in a dream state, or even presence/reality within the real world.

This view is supported by other conceptualizations of presence such as the one by Waterworth et al. (2010), who posited that presence refers to the part of the contents of our consciousness that are directly related to the current time and place in which the body is located (Waterworth et al., 2010). The authors further suggested that presence, when conceptualized in this way, is not a unitary phenomenon and likely exists on a spectrum between presence (fully engaged with the external world) and absence (fully engaged with internal cognition). This seems to be true regardless of whether such a place is real or virtual.

Recent theoretical accounts also support the idea that the psychological experience of presence far outweighs the importance of any technical factors when it comes to presence in VR specifically, calling into question why popular definitions of presence seem to be contained to VR research. Triberti et al. (2025) argued that, because VR technology operates on similar principles expected by the brain’s predictive processing mechanisms, it can “trick” the brain into enacting the same presence it feels when in “real” environments. Triberti et al. (2025) went further by adding a social dimension to their definition of presence. Instead of just being defined by agency, exteroceptive prediction, or interoceptive prediction, the authors argued that presence is, at least in part, formed from narrative context, individual-social factors, and intentional structures (Triberti et al., 2025).

For example, “culture” is often defined as patterns of representations embodied in people and contexts (Adams & Markus, 2004) that influence our shared realities. Previous research in psychology has found strong evidence that cultural backgrounds cause people to process perceptual stimuli differently (Nisbett et al., 2001; Norenzayan et al., 2002; Senzaki et al., 2014), with this research extending to objects and scenes presented to participants in VR. Šašinková et al. (2023) found that cultural background influenced the perception of complex visual scenes within VR, showing that complex visual stimuli were processed differently in VR depending on the country one grew up in (spanning Europe, Africa, and Asia within this study specifically). Although research has yet to connect such findings specifically to presence in VR, it is likely that culture will influence the feeling of “being there” in a virtual environment as much as it does in a physical one.

Additionally, social presence—the sensation of being in the company of others (Pianzola et al., 2021; Triberti et al., 2018)—is a significant phenomenon that impacts both felt reality in the real world and user presence in VR. It does not, however, seem to be instantiated differently in these different environments. For example, in both physical and virtual environments a strong sense of social presence may arise from the simple visual cue of finding footprints on the ground (Triberti et al., 2018). This provides further evidence for the idea that reality and presence are partially socially constructed, minimizing the importance of the technological aspect as the main difference between felt presence in VR and felt reality and placing emphasis instead on the psychological components of presence in even virtual environments.

Such integrative, predictive processing accounts are not the only theoretical mechanisms that can be applied to both presence in VR and presence, or reality, in the cognitive sciences. Affective realism is a theory that posits that perceptions of reality are based on affective feelings rather than any objective reality (Wormwood et al., 2019). The affective realism hypothesis states that affective feelings such as pleasure and distress are as intrinsic to our perception of the world as common physical properties such as color or brightness (Barrett & Bliss-Moreau, 2009). According to this view, objects and people in one’s environment are ascribed “positive” or “negative” affect by virtue of how one feels about them. For instance, a venomous snake may be ascribed a negative effect, whereas a pet hamster may be ascribed a positive effect.

Research has provided evidence that one’s affective experience of an object may change the perceived physical properties of that object. For example, one study found that neutral faces were perceived as more positive by participants who were exposed to smiling faces in a continuous flash suppression paradigm (Wormwood et al., 2019), whereas another study found that objects with a negative affect appeared larger than objects with a neutral or positive affect (van Ulzen et al., 2008). Because, as previously discussed, virtual environments can offer treatments utilizing exposure-based therapies (such as therapies for PTSD), there may be reason to believe that objects and people in VR are ascribed the same affective painting as objects and people in the physical environment. This is further supported by the substantial number of studies investigating the impact of VR in the treatment of phobias, which we discuss later.

Other affective accounts of presence attempt to marry the predictive frameworks developed by researchers such as Seth et al. (2012) and Berthiaume et al. (2018). This includes conceptualizations from researchers who argue that presence is an intuitive, metacognitive feeling that is generated as a result of an experience-based metacognitive judgment (e.g., Riva & Mantovani, 2012). For example, imagine you are in a virtual environment and reaching out to knock a crystal glass champagne flute off a table. You may expect two primary outcomes from such an action:

1. You feel the glass hit your hand.
2. The action of hitting the glass with your hand causes it to tip and fall off the edge of the table.

If the predicted sensorimotor outcomes generated by our embodied, intuitive simulations of the intended action are met, then one feels present within that environment. However, if the action of reaching out and knocking the glass does not result in a form of haptic feedback and/or does not cause the glass to fall from the edge of the table toward the ground, then this violates our expectations, which causes a disruption in the sense of presence. In this sense, presence is unconscious in that “we do not have detailed conscious access to its processing antecedents” (Riva & Mantovani, 2012, p. 205), conscious in that it presents a distinct and recognizable phenomenology, and metacognitive in that it conveys information regarding our conscious experience that allows us to monitor and regulate our actions.

This description of presence as a metacognitive, affective feeling mirrors other theoretical work on the sense of reality. Specifically, Dokic and Martin (2017) also conceptualized the sense of reality as “an affective experience akin to a metacognitive feeling” (p. 299) but separated this from what they termed the “sense of acquaintance”—the sense that we are acquainted with the object itself in our environments rather than a representation of that object. The authors suggested that it is possible for a one-way dissociation to occur between these two senses in that the sense of reality can occur without the corresponding sense of acquaintance. For example, it often occurs that we identify objects within virtual environments as feeling real or, as Riva and Mantovani would put it, that we have an intuitive, metacognitive affective feeling that they are real without necessarily having a sense of acquaintance with said object (e.g., we cannot feel it when our virtual hands touch it).

Similar to the cases of mechanisms presented by Seth et al. (2012) and Berthiaume et al. (2018), these affective theories regarding presence and reality seem to have been developed independently of one another despite their significant similarities, further going to show how the concepts of “reality” and “presence” are likely to be far closer both by definition and mechanistically than it may immediately appear.

## Consolidation of Psychophysical Mechanisms and Their Supportive Evidence Across the Fields

Beyond integrative theoretical accounts, studies investigating both presence and reality have uncovered similar findings using psychophysical methods, demonstrating a significant overlap of converging evidence between these fields. As summarized in Table 1, key mechanisms such as predictive processing, multisensory integration, self-embodiment, and shared neural substrates show striking parallels in the findings of both presence and reality research. We elaborate on these points of convergence in the paragraphs that follow, arguing that they provide further evidence for a continuous mechanism underlying the senses of presence and reality.

**Table 1.** Summary of Converging Evidence for Continuous Neural and Psychophysical Mechanisms Underlying Presence and Reality

Mechanism	VR/presence research	Physical world/reality research
Predictive processing	EEG studies have found evidence of repetition suppression—a neural marker of predictive processing—in response to repeated stimuli in VR environments. Theoretical models posit that presence is modulated by prediction errors arising from mismatches between expected and actual sensory input.	The experience of reality is generated by the brain minimizing prediction errors between its internal models and incoming sensory data. fMRI studies show repetition suppression in response to expected stimuli, providing key empirical support for this framework.
Multisensory integration	The presentation of incongruent multisensory stimuli in a virtual environment has been found to negatively impact the user’s reported sense of presence.	Violating expectations by pairing incongruent sensory stimuli in non-VR settings induces feelings of body disownership, which are characteristic symptoms of DPDR.
Self-embodiment and agency	VR can induce powerful full-body transfer illusions, causing participants to experience a virtual body as their own that, in turn, improves presence.	The disruption of agency and self-embodiment through, for example, the rubber-hand illusion, has been shown to increase symptoms of dissociation in clinical populations with PTSD.
Perspective-taking	Placing participants in a third-person perspective within a VR environment has been shown to induce temporary dissociative symptoms in healthy subjects.	A first-person, embodied perspective is considered crucial for a stable sense of reality. Disorders of body ownership (e.g., somatoparaphrenia) demonstrate a marked dissociation between first- and third-person experiences of selfhood.
Neural substrates	Neuroimaging studies have found that the sense of presence is significantly correlated with activity in brain regions, including the dorsolateral prefrontal cortex, the insula, and the amygdala.	Disorders of reality, such as DPDR, are associated with dysregulation in a similar frontolimbic network that includes the prefrontal cortex, insula, and amygdala.

Note: VR = virtual reality; fMRI = functional MRI; DPDR = depersonalization-derealization disorder; PTSD = posttraumatic stress disorder.

As explained in detail in the previous section, fields of both presence and reality place strong emphasis on the importance of prediction and expectation of incoming perceptual information. Although such predictive processing models have been criticized because of a lack of empirical support caused by considerable methodological challenges (Walsh et al., 2020), there is some limited empirical evidence that supports such a unifying, predictive account of neural functioning. Repetition suppression is a phenomenon in the field of external perception in which a repeated stimulus presented to a participant causes a priming-related decrease in neuronal activity (Auksztulewicz & Friston, 2016). In the context of predictive processing theories, this occurs because the prediction error arising from a repeated stimulus is minimized because it becomes expected, reducing neuronal activity relating to that stimulus. Utilizing functional MRI (fMRI), studies have shown that these reductions are typically observed within the primary sensory cortex (Tang et al., 2018; Wacongne et al., 2011). However, violations of more complex regularities have been shown to evoke activity in brain regions typically associated with higher level cognitive functioning, such as the temporal lobe and frontal cortex (Dürschmid et al., 2016; Heilbron & Chait, 2018).

Similar evidence for perceptual predictive processing has been observed in virtual environments. Studies utilizing VR have used EEG to show that repeated visual stimulation in VR has a similar impact of repetition suppression on neural response as in typical, “real-world” studies (Johnsdorf et al., 2023; Kisker et al., 2024). Although studies utilizing VR have so far not been used to spatially locate the region of such neural suppression, we nevertheless take the convergence of these results to indicate that similar predictive mechanisms used in the perception of real-world external phenomena are also used in the perception of virtual phenomena.

Similar converging evidence has been uncovered in the field of multisensory integration, an area of particular importance for both reality perception and perception in VR. Multisensory integration is the process by which incoming perceptual information from different modalities is combined into one percept (Cornelio et al., 2021; Deroy et al., 2016). Multisensory integration is vital in both presence and reality because it allows for the formation of not only judgments of perceptual coherence of external percepts but also a coherent perception of one’s own body in space (Ehrsson, 2012). Multisensory integration is also a clear example of how mechanisms of predictive processing could be applied both to reality and VR environments.

Previous studies in the sense of reality have shown that violating expectations in multimodal conditions by pairing incongruent sensory stimuli induces a reduction in bodily self-consciousness and agency with an increase in feelings of body disownership (Fotopoulou et al., 2011; Jenkinson et al., 2022; Valzolgher et al., 2018). Such alterations, in particular feelings of body disownership, are consistent with what we might consider symptoms of unreality that are characteristic of DPDR.

DPDR is characterized as a disorder in which patients describe the external world and/or their own bodies as feeling unreal, distant, or distorted (Yang et al., 2023) and is an example of an errant sense of reality. The prevalence of these disorders has been suggested to be somewhere between 0% and 1.9% of the general population (Yang et al., 2023), with diagnosis often assessed by comparing patient symptoms to diagnostic criteria in the the fifth edition of the *Diagnostic and Statistical Manual of Mental Disorders* (American Psychiatric Association, 2013). Thus, we can infer that congruent multisensory experiences are crucial in the stable perception of reality because prediction errors arising from a mismatch between expected multisensory congruency and incoming multimodal perceptual information induce unreal-like experiences. Similarly, previous studies investigating the sense of presence found that the presentation of incongruent multisensory stimuli negatively impacted presence, whereas congruent multisensory stimuli increased presence (Kim & Lee, 2022). This mirrors findings from non-VR studies, suggesting that multisensory integration plays a similarly significant role in both maintaining the perception of presence and reality.

This importance of multisensory integration on presence and reality may be, at least in part, due to the shared importance of interoceptive signals. In the previous section, we highlighted the importance of the prediction of both exteroceptive and interoceptive signals on the sense of presence and reality in the theoretical conceptualizations proposed by Seth et al. (2012) and Pianzola et al. (2021). However, previous research has found that incoming interoceptive signals are able to directly influence the experience of body ownership via multisensory integration—specifically in the way in which selfhood emerges from the integration between interoceptive representations and exteroceptive signals (Suzuki et al., 2013).

Suzuki et al. (2013) found that synchronous cardiovisual feedback led to an enhanced experience of ownership in the rubber-hand illusion (RHI) compared with asynchronous feedback. Additionally, results from an RHI experiment by Crucianelli et al. (2018) suggested that the perception of interoceptive signals and their impacts on feelings of body ownership depended on an individual’s ability to regulate the balance of interoceptive and exteroceptive signals. Furthermore, another RHI study from Filippetti et al. (2019) found that incongruency between the visual aspect of being touched by a fabric and the texture of the fabric led to a reduction in feelings of embodiment over and above temporal and spatial properties of multisensory integration (Filippetti et al., 2019). Such findings support the idea that the roles of multisensory integration and interoceptive prediction are linked to feelings of body ownership. Because a first-person, embodied perspective is vital for the successful instantiation of presence and reality (as we discuss later in this section), this increase in feelings of body ownership is likely linked to increases in both feelings of presence and reality.

Multisensory integration also plays a role in concepts of agency and self-embodiment. Research on agency and self-embodiment has found that loss of agency over one’s body and motor actions can induce and worsen symptoms of DPDR. Agency and self-embodiment, in the contexts of predictive processing models of presence and reality, would be considered as higher order top-down modulators of incoming perceptual information. One study conducted by Rabellino et al. (2018) used the RHI to determine whether patients with a dissociative subtype of PTSD (PTSD that causes DPDR-like experiences) experienced greater symptoms of dissociation when their agency and self-embodiment were disrupted by the RHI. The authors found that participants did feel greater dissociation during the RHI (Rabellino et al., 2018). This provides some evidence that a stable sense of agency and self-embodiment is important in maintaining a coherent sense of reality.

Further to this point, Clode et al. (2024) developed a hand-augmentation device nicknamed the “third thumb” that was controlled by pressure sensors in the shoe of participants. The authors found that, despite the seemingly odd setup of controlling a prosthetic thumb with movements of the big toe, 98% of participants were successfully able to wear, operate, and perform tasks with the prosthetic device (Clode et al., 2024). Furthermore, participants reported that after training with the third thumb they felt it was now a part of their body.

The field of presence has uncovered similar findings. VR has been shown to induce full-body transfer illusions, similar in nature to the rubber-hand and sixth-finger illusions, in which participants experience virtual bodies as though they are their own despite the higher order knowledge that they are in a virtual environment. One study found that a multisensory visuohaptic first-person perspective of a life-size female body in VR was enough to induce a full-body transfer illusion in male participants (Slater et al., 2010), who reported feeling the virtual body as if it were their own. The authors concluded that their results support the notion that bottom-up perceptual mechanisms are sufficient to override top-down knowledge of reality, similar to potential mechanisms regarding the six-finger illusion. These results seem to suggest that predictive processing in both VR and reality allows for prior expectations about the self and the world to be overridden by sustained prediction errors.

Last, as mentioned previously, the disruption of the sense of reality can result in DPDR. Although these types of dissociation are assumed to be induced clinically by life traumas or other comorbid mental-health disorders such as anxiety or depression (Yang et al., 2023), experimental research has found that acute dissociations from both the body and environment can be induced in the lab through VR manipulation. This is a significant finding because it provides evidence that manipulations of perception in VR are sufficient to produce what would normally be considered disorders of reality perception, suggesting that reality and presence are not as siloed as some researchers may consider them to be.

For example, van Heugten-van der Kloet et al. (2018) succeeded in inducing temporary dissociative symptomatology in healthy subjects. Participants were placed in a virtual environment in which they were looking down on themselves in the third person and asked to complete various tasks. This perspective was designed to induce as little presence in VR as possible by virtually removing participants’ perspectives away from their own bodies and actions. Participants reported a large, significant increase in acute dissociation post-VRET (van Heugten-van der Kloet et al., 2018), highlighting the ability of the exposure to an ultralow-presence environment to induce dissociative symptoms in otherwise clinically healthy adults. This is in line with research from other domains that has found that, although a minority of participants do spontaneously choose to take on decentered perspectives in an object-recognition task, forcing a participant who prefers to view an object from a self-centered perspective to view it from a decentered perspective results in a significant reduction in task performance (Arnold et al., 2016).

Such findings support the concept of both the sense of presence and the sense of reality being phenomena that rely on a first-person, embodied perspective of the surrounding environment (Fotopoulou et al., 2011; Riva & Mantovani, 2012). If a person is unable to experience themselves as an agent from the first-person point of view, it seems as though both the sense of presence and reality are disrupted, producing similar errant phenomenology. Such results support theoretical conceptualizations of presence such as the one by Riva and Mantovani (2012) noted in the previous section in which they describe presence as a metacognitive feeling driven by the fulfillment of predicted action from an embodied simulation. These findings are also supported by research from the field of somatoparaphrenia research that has found dissociations between third- and first-person feelings of body ownership. Patients suffering from somatoparaphrenia—the feeling that parts of one’s body do not belong to oneself—seem to reverse these feelings of otherness if they view themselves from a third-person perspective in a mirror. However, this reversal does not last, and feelings of otherness still return when the mirror is removed (Fotopoulou et al., 2011). Such a marked dissociation between experiences of selfhood in the third and first person mirrors the dissociation between the experiences of presence in the third and first person.

Taken altogether, it appears that there are key processes involved in the sense of both presence and reality, such as the role of prediction and expectation, the role of multisensory integration, the role of agency, and the role of self-embodiment, with each of these holding similar importance. Although this in itself does not necessarily mean that presence and reality share mechanisms, we nevertheless take it as further evidence for such a conclusion considering the lack of direct empirical comparison.

## Consolidation of Neural Mechanisms and Their Supportive Evidence Across the Fields

Although little is known about the neurobiological underpinnings of either the sense of presence or the sense of reality, both have been subject to a limited number of studies using neuroimaging techniques. In particular, fMRI has been used to attempt to localize the sense of presence as well as disorders of reality perception such as DPDR to brain regions. Once again, the findings of these studies seem to highlight the importance of identical brain regions, and we take this as some limited further evidence of shared mechanisms between reality and presence.

Although investigations into the brain regions associated with DPDR are preliminary, previous fMRI studies have identified various brain regions as being associated with the disorder. For example, Phillips et al. (2001) showed increased activity in prefrontal cortical regions such as the ventral prefrontal cortex and the anterior cingulate alongside decreased limbic activation in the amygdala and hypothalamus, with other studies specifically identifying these regions as the right amygdala and right hypothalamus (Lemche et al., 2016; Medford et al., 2016). It is thought that these feelings of unfamiliarity within oneself and the surrounding environment may be explained by a frontolimbic inhibition model of DPDR (Murphy, 2023) in which limbic regions are abnormally inhibited by top-down control of the prefrontal cortex. In terms of a predictive processing framework of presence and reality, this could be framed as the aberrant suppression of bottom-up limbic signals by an overactive prefrontal cortex projecting overweighted top-down predictions. These results imply that a well-functioning sense of reality is regulated by balanced activity in the prefrontal cortex, insula, right amygdala, and right hypothalamus.

Neuroimaging research on the sense of presence has come to similar conclusions regarding brain region localization. Studies have found activity of the dorsolateral prefrontal cortex (Baumgartner et al., 2008; Clemente et al., 2014), insula and postcentral parietal cortex (Clemente et al., 2014), and the amygdala (Ogawa et al., 2013) to be significantly correlated with the sense of presence. Although there is no formal frontolimbic model of the sense of presence, these results may indicate that the same balance of activity maintained between the prefrontal cortex, insula, and amygdala crucial for the maintenance of presence may be disrupted to cause DPDR symptoms outside of VR.

It is important to point out that although similarities between brain regions responsible for the sense of reality and the sense of presence are interesting, by themselves they do not suggest any causality between the two. However, we believe that when considered within the wider context of our argument, this provides further, indirect evidence that distinctions between reality and presence are unnecessary.

## Potential Limitations of This Account and Unanswered Questions

One potential limitation to our argument is that, despite similarities between integrative mechanisms of presence and reality, there may be some top-down modulation of incoming perceptual information in VR that renders it sufficiently distinct from incoming perceptual information from the physical world. This may meaningfully alter the subjective interpretation of perception in VR, rendering it distinct from reality perception of the physical environment and meaning that conclusions regarding presence from VR research cannot necessarily be extrapolated to presence (or reality) in cognitive science. Although we accept that such top-down modulation of incoming perceptual information can, and often will, occur in the form of a Bayesian/predictive account of top-down expectation of incoming perceptual information (Clark, 2013; Friston et al., 2006, 2015), there currently is no evidence to suggest that priors regarding the expectation of incoming perceptual information differ between reality and VR. In fact, evidence suggests that expectations typically associated with real-world experience are violated in VR, such as multisensory incongruencies or lack of haptic feedback, which markedly reduces presence (Kim & Lee, 2022) and, in extreme scenarios, induces DPDR-like experiences (van Heugten-van der Kloet et al., 2018). This suggests that although top-down processing influences perception at the level of immediate perceptual consciousness, the priors driving such expectations are identical between the physical world and VR.

A second limitation of this account is that presence may be a subset, or a special case, of reality perception as opposed to a fully continuous concept. Suzuki et al. (2023), in particular, argued that presence is a subset of what could be considered the sense of reality (Suzuki et al., 2023). Although the authors agreed that most of the literature involved in the debate around the sense of reality is focused on concepts that one could consider “presence,” the concept of “presence” itself ignores what the authors termed “doxastic veridicality”—a term from philosophy and linguistics that refers to whether a person’s belief or other mental state (doxastic state) accurately reflects reality (veridicality; e.g., “I believe this keyboard I am typing on really exists” has high doxastic veridicality). Because very few clinically healthy individuals would consider a virtual environment as being part of the real world, presence therefore does not fulfill this condition of the sense of reality.

However, we believe that Suzuki et al. (2023) may have overstated the necessity of doxastic veridicality in defining the sense of reality. We see repeatedly in literature covering hallucinations, presence, VR, and DPDR that experiential reality is largely independent of the knowledge of whether such an experience is taking place within the real world. DPDR patients, for example, know that they are not actually outside of their own bodies and that the world around them is real, yet their experience of the world is one of intense unrealness. The same can be said for common optical illusions. No one believes that static images of colored geometric shapes are really moving, yet we undeniably experience them as such. Similarly, in VR, participants maintain the doxastic understanding that their current environments are not situated in the real world, yet they often behaviorally and physiologically react to events as if they are. This lack of doxastic veridicality does not seem to impact the experiential nature of reality or presence.

Another objection against our argument concerns the differential impact of traumatic situations presented to people in VR versus real life. In real life, trauma is often defined by a total loss of control of external events that can oftentimes have very real and permanent lasting impacts on the person experiencing them. For example, traumatic events experienced in real life such as experience in military combat zones can typically result in PTSD for soldiers returning home. In a review, Richardson et al. (2010) found that between 4% and 17% of U.S. Iraq War veterans experienced combat-related PTSD (Richardson et al., 2010). First-person shooter games that simulate war are some of the most popular types of games to play in VR. However, although data on this particular topic are scarce, we believe it is unlikely that PTSD occurrences in people who regularly play such games match the data from Richardson et al. (2010).

This may be for a number of reasons. First, it may be that self-selection among users means that individuals who believe they may be traumatized by such an experience would simply avoid having such experiences in VR. This would result in far fewer cases of PTSD from virtual experiences than otherwise expected. However, the same self-selection could be said of putting oneself in a real-life combat situation, in that one who believes they would be traumatized by such an experience would go out of their way to avoid it. However, this explanation ignores the role of various socioeconomic pressures (Bennett & McDonald, 2013) as well as propaganda in one’s decision to enroll in a military force.

A second reason may be that there are limits to the intensity, realism, or chronicity of typical VR content available to users that protect them from the risk of long-term trauma. We have previously argued and go on to argue further in this article that this lack of intensity and realism is not because VR is incapable of producing intense and realistic experiences, but because the creation and propagation of such experiences are not desirable to companies producing VR headsets or to videogame developers whose primary goal is to sell as many units of their games as possible. Although it is, of course, unethical to attempt to induce long-term psychological trauma such as PTSD in participants experimentally via VR, studies have been done that have attempted to investigate the experience of horror video games in VR and their impact on the psyche. In a review, Lin (2023) found that participants engaged in self-help coping strategies when completing VR horror games similarly to how one might cope with stress-inducing situations in real life. Active approach strategies, constant self-reminders that their environment was only virtual, and disengaging from situations both mentally and physically were among the most common reported strategies (Lin, 2023). The presence of such behavior, even in a VR simulation deemed safe and ethical enough for general and experimental use, may in fact demonstrate the potential damage of VR experiences specifically designed to induce trauma in users.

The results of such experiments also contradict a third possible reason why VR does not seem to induce trauma in users: Virtual events are not beyond the control of the person experiencing them. The knowledge that one is in a simulated environment, regardless of whether this knowledge is conscious or unconscious or indeed attended to during the moment of the VR experience, might function as a type of cognitive shield that may prevent the brain from encoding such an experience as real, life-threatening trauma. Although the participants tried to implement this kind of shield in the form of reminding themselves that their environment was only virtual (Lin, 2023), certain participants still showed residual fear even a day after having played the game. A study by Lin (2017) investigated fear elements, coping reactions, and immediate and next-day fright responses of participants to a zombie VR game. Here, the author found that, again, certain participants experienced next-day fright after playing the game that consisted of cognitive reactions and a lasting fear of being attacked from behind (Lin, 2017). A different study by Lavoie et al. (2021) found that participants who were exposed to a scenario in VR that was designed to elicit low to moderate amounts of negative emotion when administered outside VR experienced intensified negative emotional responses that were positively correlated with self-reported feelings of negative rumination (Lavoie et al., 2021). Although, admittedly, none of these studies investigated VR-related PTSD or other long-term trauma induced by virtual events, perhaps the omission of such an investigation was more due to ethical concerns than to any lack of theoretical merit.

A further reason that may explain the lack of long-term trauma induced by VR experiences is the absence of real bodily harm or objective threat. Because these experiences take place in a virtual environment, it does not matter how psychologically traumatizing such an environment may be—it will never result in lasting physical harm to the body. However, although this is objectively correct, there have been some case studies that suggest that the impossibility of physical harm resulting from a virtual experience does not entirely protect the psyche of the user from long-term trauma. There have been reports and case studies of individuals developing forms of trauma from events that took place in VR—specifically from cases of virtual sexual assault. Women who have participated in social VR online experiences, such as VRChat or Horizon Worlds, have reported experiencing their avatar being trapped in a virtual room and subjected to violent or sexual-assault behaviors by one or multiple assailants (Porta et al., 2024). In one case in 2024, British police opened a criminal investigation into the virtual gang rape of a 16-year-old girl on Horizon Worlds by a group of strangers (Sales, 2024). In their investigation, police asserted that the psychological trauma inflicted on the girl was “similar to that of someone who has been physically raped” (Camber, 2024, para. 12).

Although we do not have access to official psychologists’ reports, such case studies and reports provide some emerging evidence that VR interactions hold the potential to induce traumatic experiences in certain individuals. Nevertheless, it is unknown whether the mechanisms causing such trauma are associated with the vivid perceptual aspects of VR, the psychology of the individual experiencing the event within VR, or a mixture of the two. However, the psychology of trauma is an area with a wealth of literature suggesting that risk factors such as an individual’s fear of lack of control or agency may contribute heavily to trauma-related distress (Ataria, 2015; Gershuny & Thayer, 1999). It is therefore likely that such trauma in VR arises from similar mechanisms rather than being influenced by the virtual medium per se.

If presence in VR and the sense of reality were largely separate phenomena, then we may not expect exposure to the same traumatic events in VR as one has experienced in a physical environment to provide any sort of substitute for traditional exposure therapy. However, this is not the case. VR has already been investigated as a potential tool for exposure therapy for certain anxiety disorders, such as panic disorder (Botella et al., 2007), social phobia (Klinger et al., 2005), PTSD (Difede & Cukor, 2007), fear of flying (Rothbaum et al., 2006), fear of spiders (Carlin et al., 1997; Garcia-Palacios et al., 2002), fear of heights (Krijn et al., 2004), and psychosis (Rus-Calafell et al., 2018)—substituting real-life exposure for a more manageable virtual exposure. All of these studies reported a significant improvement of symptoms post-VRET, demonstrating its effectiveness in reducing domain-specific distress and general subjective stress in cognitive, behavioral, and psychophysiological measures (Powers & Emmelkamp, 2008).

VRET has even been shown to be an effective treatment for PTSD. A meta-analysis conducted by Kothgassner et al. (2019) found that VRET may be equally as effective as active comparators for PTSD patients. In other words, there is no difference in outcomes between exposure therapies for PTSD patients undertaken in VR and exposure therapies undertaken in the physical world (Kothgassner et al., 2019). This same conclusion was drawn in a later meta-analysis conducted by Eshuis et al (2021). In all of these cases of phobias and PTSD, exposure to a controlled virtual environment designed to mimic triggering scenarios produced a long-lasting therapeutic effect (Eshuis et al., 2021). Therefore, even though a participant recognizes that their environment is not real, experiences in VR seem to be enough to induce long-term psychological improvement via exposure to triggering situations. Although this is consistent with our integrative view that presence and reality are continuous, it does not by itself prove any particular mechanistic account of presence or reality. The implication here that because VR can provide therapeutic benefits for patients with long-term trauma that it also has the theoretical capacity to induce long-term trauma is purely speculative because there is an absence of literature on this topic.

Additionally, research comparing the efficacy of VRET to prolonged exposure (PE) techniques that typically involve a mixture of imaginal exposure and in vivo exposure therapies has uncovered a variety of findings. First, it appears that VRET is equally as effective as PE in the treatment of PTSD (Difede et al., 2022; Gonçalves et al., 2012), which may seem surprising considering that one might expect VRET to be more effective than a medium that involves simply imagining being in the threatening environment. However, additional research has shown that some patients display a greater reduction in PTSD symptoms when undergoing PE (Reger et al., 2016; Norr et al., 2018), whereas others experience a greater reduction when undergoing VRET (Norr et al., 2018). One study suggested that patients with a greater reduction in symptoms via VRET were likely to be younger, to not be taking antidepressant medication, and to have greater PTSD hyperarousal symptoms as well as a greater than minimal suicide risk (Norr et al., 2018). Taken together, although it seems that there is no difference between PE and VRET outcomes for PTSD patients, their efficacy does differ depending on the specific demographics and symptomatology of that particular patient’s PTSD, making it difficult to determine the exact role of presence when it comes to treating the disorder.

If presence and reality were separate phenomena, then we might expect VRET to have a similar impact on a patient’s well-being as looking at pictures or videos of triggering situations. However, although research investigating VRET directly against video-based therapies is slim, limited existing studies suggest that this may not be the case. One study conducted by Tortella-Feliu et al. (2011) compared the efficacy of VRET to computer-aided exposure with a therapist (CAE-T) to self-administered computer-aided exposure (CAE-SA). In the behavioral analysis, the authors found that 78.9% of the VRET group completed a flight 2 weeks after the final treatment session compared with 61.9% in the CAE-SA group and only 50% in the CAE-T group (Tortella-Feliu et al., 2011). However, there are admittedly many other factors that differentiate VRET from CAE-T and CAE-SA therapies beyond the addition of presence. In VR, participants are typically embodied and have greater agency over their own actions, which is supported by interactivity with their environments. These are all factors that influence the sense of presence, but because, like presence, they are not things typically present in CAE-T and CAE-SA therapies, we cannot definitively conclude that VRET is a superior treatment option solely because of the role of presence. Additionally, Tortella-Feliu et al. (2011) did not report that such differences in outcomes between treatment strategies were statistically significant, although we suspect this was largely because of the small sample size of the study (20 per condition). If similar descriptive trends were replicated in a larger, more well-powered trial then this would be stronger evidence for presence as being a contributing factor in the efficacy of VRET compared with other types of therapy. Although not conclusive, this does provide at least preliminary evidence that there is a separation between the efficacy of video-based treatments compared with VRET treatments in psychological disorders, and we theorize that this difference is at least partly caused by the sense of presence.

It remains to be seen whether purely virtual experiences may be enough to induce long-lasting psychological trauma in participants, although as adoption to such technology slowly increases and more of people’s time is spent in virtual spaces, we would be unsurprised to learn that this comes with an increased risk of long-term psychological effects. For example, there have been documented cases of films inducing serious psychological trauma on consumers, including one case study in which a young woman suffered from intrusive thoughts and flashbacks to events depicted in the film *The Exorcist* (Ballon & Leszcz, 2007). Such cases have been termed “cinematic neurosis”—a form of psychological crisis that is shaped by events that have taken place in a film. As previously discussed, video as a medium does not seem to have the same capacity to influence the human psyche as VR, which induces a sense of presence and embodiment within a virtual space. The lack of similar cases of cinematic neurosis reported coming from VR environments is perhaps less because of a cognitive shield effect and more because VR hardware is in its infancy when it comes to widespread adoption by regular users.

A final, unanswered question is why exactly so many researchers studying presence in VR refer to presence as something that must be driven by higher order cognitive mechanisms. Such researchers will often purport to be investigating presence at the perceptual level (e.g., the impact of visual fidelity in VR on presence) yet define and discuss presence as if it were something driven by higher order thought. We suspect that it is possible that the conceptualization of presence as something that must necessarily occur in VR among VR researchers has proliferated so heavily because of the nature of the dependent variables of most presence research.

In most studies, presence is measured using the Witmer-Singer presence questionnaire (Witmer & Singer, 1998), IPQ (Tran et al., 2024), ITC–Sense of Presence Inventory (Lessiter et al., 2001), or Slater-Usoh-Steed questionnaire (Usoh et al., 2000) administered after each block of an experiment. Questionnaires are often used to measure participant presence because of their affordability and ease of administration while still producing broadly replicable results. It is not necessarily incorrect to study presence using such measures. As we have discussed, presence is likely to occur both at the perceptual level and at the cognitive level. However, such questionnaire methods are more likely to capture measures of presence at the cognitive level rather than the perceptual level.

Post hoc measures of immediate perceptual phenomena have been criticized for decades (Blankenburg, 1971), with some philosophers arguing that post hoc judgments of the subjective experience of immediate perceptual phenomena are entirely distinct measures from the immediate subjective experience itself (Jaspers, 1913). Therefore, the prevalence of questionnaire usage in presence literature may have introduced an unintentional bias. Specifically, researchers may unintentionally view presence as having been primarily created by a higher cognitive process because such a cognitive process is required to report one’s prior levels of presence in a questionnaire format administered after a VR experiment. This creates a confusion in which researchers who investigate perceptual models of presence unintentionally refer to presence as a cognitive process because generating the dependent variable of their study relies on such a cognitive process. This is part of the reason why many modern methods of measuring presence in participants are beginning to find avenues to move away from self-report, preferring to instead utilize electrophysiological and behavioral measures of presence such as skin conductance, heart rate, and reaction time (Grassini & Laumann, 2020).

## Presence and Reality in Other Environments

There are other examples of environments in which one might report feeling a sense of presence and reality despite the fact that those environments are neither “real” nor exactly “virtual” in the way we have been describing so far. These are: dreams, vivid mind wandering (specifically intrusive imagery), and phenomena related to psychotic delusions. We argue here that presence and reality within these environments is consistent with our conception of presence and reality as consistent phenomena.

As mentioned previously, there is no reason to believe that the presence that one feels when one is dreaming is any different from the presence one feels in a real environment or a virtual one. Dreams are virtually externalized (Revonsuo, 2006), allowing the perceiver to feel as if the dream is taking place within the real world, similar to how VR systems externalize environments to allow one to feel as if the experience is taking place within a real space. Even if the dreamer is lucid and able to recognize that their current experience is taking place within a false environment, there is no evidence to show that this reduces their sense of presence within that false world. If anything, it is far easier to create a sense of presence in a dream because one does not remember the process of falling asleep, unlike the process of putting on a VR headset. Even though one is able to recognize that the dream did not take place within the real environment after waking up, this does not change the fact that, in that moment, they felt entirely present. This dissociation reflects a difference between the immediate, prereflective “felt reality” (presence) and a later metacognitive reality judgment.

Waking states in which the brain utilizes imaginative processes, such as vivid mind wandering and intrusive imagery, act on a similar principle. In the case of intrusive imagery, for example, at the moment of perception, one might have a sudden and involuntary mental image of a distressing event, often accompanied by sounds or physical sensations (Brewin et al., 2010). Intrusive imagery differs from hallucinations in that patients will not report that they physically see the event externally within their surrounding environment but in their mind. Importantly, this does not stop patients from reporting that such events feel real, which may superficially appear as a case of a sense of reality with no sense of presence. However, we theorize that there is still a false externality here in the sense that the event is still taking place within a virtual, mental environment rather than a physical one (similar to a dream). The event still feels real and present because of this virtual externalization despite the fact that the physical external environment remains unchanged.

Psychotic delusions and other related phenomena, particularly in the cases of nonvisual delusions, are also examples in which presence and reality converge. Patients with psychosis may show symptoms such as thought insertion, a delusion in which an individual believes that thoughts are being inserted into their minds through an outside source (López-Silva, 2018), such as a microchip in their head or from angelic or demonic beings. In such cases, patients can be said to have a deep sense of reality regarding these delusions, hence the difficulty in treating such conditions (López-Silva, 2018). However, we believe there is also a presence regarding these symptoms despite the typically weak sensory qualities of thought insertion. In the case of a microchip, the patient believes that thoughts are being inserted into their minds via a microchip that is actively present within their brain. In the case of angels or demons, the patient believes that the beings inserting thoughts into their mind are present within the environment around them without actually being able to otherwise see or interact with them. In this way, neither presence nor reality are necessarily reliant on exteroceptive sensory signals for their manifestation, in line with the model laid out by Pianzola et al. (2021).

## Concluding Remarks, Implications, and Further Investigations

In summary, the sense of presence and sense of reality, although widely defined and used by researchers in their respective fields as distinct phenomena (which nevertheless are still often interchangeably used), can be considered conceptually, mechanistically, and phenomenologically continuous. The idea that presence in VR is distinct from presence or reality in cognitive science is born from a combination of artificially separated definitions and a misunderstanding of the impact of top-down knowledge of the virtual nature of one’s environment. This leads to (or perhaps results from) an artificial dichotomizing of perceptual versus cognitive accounts of presence in VR. Once we understand presence as an integrative process and combine this understanding with converging evidence from psychophysical and neuroimaging findings, we can see that the sense of presence and the sense of reality are continuous.

A new definition of presence in VR research can be more accurately defined as identical to the definition of the sense of reality and presence in cognitive science in that feeling as if you/your environment is present (or real) is different from feeling as if you/your environment is not present (or unreal). Converging evidence from both fields only goes further to suggest that these concepts are far more likely to be shared than distinct processes. Indeed, additional preliminary neurological evidence may support this hypothesis by highlighting shared frontolimbic mechanisms between presence and reality.

If senses of presence and reality are indeed continuous, then the results of presence research typically done in VR can be extrapolated to how one experiences and constructs the sense of reality. Preliminary studies have already begun this process. Drori et al. (2020) used a VR environment to investigate how stable the sense of reality is as a psychometric property of experience by slightly altering aspects of the environment to induce DPDR sensations. The authors concluded that their VR methodology to measure the sense of reality was ecologically valid, potentially allowing them to utilize such a methodology as a diagnostic and therapeutic intervention for DPDR (Drori et al., 2020). We support such a conclusion, and we believe that the incorporation of VR technology and methodologies classically used in the field of presence provides a wealth of opportunity in the study, diagnostics, and intervention for disorders of reality such as DPDR.

Fully incorporating VR research on presence into the sense of reality could have far wider implications. As previously discussed, VR has already been investigated as a potential tool for exposure therapy for certain psychological disorders, with research broadly finding that VR environments are not only safe, controlled ways to practice exposure therapy but also that they produce long-lasting psychological impacts comparable to in vivo exposure therapy (Eshuis et al., 2021; Kothgassner et al., 2019). However, although adoption of such practices in real-world therapies is growing, it is far from widespread. A recent study by Felnhofer et al. (2025) found that among 694 clinical psychologists and psychotherapists surveyed, only 1.4% reported using therapeutic VR despite 20.5% reporting an interest in adopting its use (Felnhofer et al., 2025).

Common themes for this lack of adoption among even interested therapists were a lack of time to train using the equipment, a lack of access to the equipment itself, and concerns surrounding the clinical applicability and the immaturity of the technology (Felnhofer et al., 2025). Although researchers have limited powers to improve clinical access to VR systems, it is clear that more can be done not only to provide resources for training for interested therapists but also to highlight the potential benefits of VR in therapy—especially because potential drawbacks of the substitution of real stressors for virtual stressors would be minimal.

Last, the idea that presence and reality can overlap both phenomenologically and mechanistically has important implications for philosophy and cognitive science. This challenges traditional distinctions between the “real” and the “virtual” and provides further support for the idea that our experience of reality may not be an absolute, objective reality but a construct that is dynamically shaped by both the current perceptual experience and top-down factors about the context of this experience and the individual’s history and prior experience. Such a perspective frames experiences in virtual environments not as separate, isolated constructions that require their own mechanisms to understand but as experiences that are continuous with the way in which we perceive reality in the physical world.

Although we hold this position, we do acknowledge that having different terms to describe the feeling of being within a virtual environment and a real environment may be useful, if only for the sake of pragmatics. For example, using “presence” to describe the feeling of being in a virtual environment and “reality” to describe the feeling of being in a physical environment, despite their mechanistic and phenomenological similarities, would be an entirely sensible solution. Our major issue with current usages of the terms is that they are not consistent in this way.

One of the greatest drawbacks of our current argument is that, to our knowledge, no study has been conducted that has attempted to identify similarities or differences in a direct comparison between the experience of the sense of reality or the sense of presence. It is therefore important that future research attempts to investigate this hypothesis further by directly comparing mechanisms of presence and reality to determine whether they follow the same principles. For example, a future study may attempt to determine whether disrupting sensory congruence impacts presence in the same way and in the same magnitude as sensory incongruence induces symptoms of DPDR—investigating the impact of both sensory incongruence and violations of expectation on reality and presence simultaneously. Or a future study may attempt to consolidate similarities in the neurological underpinnings of presence and reality by determining whether disruptions to presence within VR cause similar impacts on the frontolimbic functioning of subjects as it does in patients suffering with DPDR. Regardless of methodology, it is only by direct comparison over a multitude of studies that such a radical hypothesis can be fully investigated.

## Transparency

*Action Editor:* Zhicheng Lin

*Editor:* Arturo E. Hernandez

## References

[bibr1-17456916251414030] Adams G. Markus H. R. (2004). Toward a conception of culture suitable for a social psychology of culture. In Schaller M. Crandall C. S. (Eds.), The psychological foundations of culture (pp. 335–360). Lawrence Erlbaum Associates.

[bibr2-17456916251414030] American Psychiatric Association. (2013). Diagnostic and statistical manual of mental disorders (5th ed.).

[bibr3-17456916251414030] Arnold G. Spence C. Auvray M. (2016). Taking someone else’s spatial perspective: Natural stance or effortful decentring? Cognition, 148, 27–33. 10.1016/j.cognition.2015.12.006 26722709

[bibr4-17456916251414030] Ataria Y. (2015). Sense of ownership and sense of agency during trauma. Phenomenology and the Cognitive Sciences, 14, 199–212. 10.1007/s11097-013-9334-y

[bibr5-17456916251414030] Auksztulewicz R. Friston K. (2016). Repetition suppression and its contextual determinants in predictive coding. Cortex, 80, 125–140. 10.1016/j.cortex.2015.11.024 26861557 PMC5405056

[bibr6-17456916251414030] Ballon B. Leszcz M. (2007). Horror films: Tales to master terror or shapers of trauma? American Journal of Psychotherapy, 61(2), 211–230. 10.1176/appi.psychotherapy.2007.61.2.211 17760323

[bibr7-17456916251414030] Barranco Merino R. Higuera-Trujillo J. L. Llinares Millán C . (2023). The use of sense of presence in studies on human behavior in virtual environments: A systematic review. Applied Sciences, 13(24), Article 13095. 10.3390/app132413095

[bibr8-17456916251414030] Barrett L. F. Bliss-Moreau E. (2009). Affect as a psychological primitive. In Zanna M. P. (Ed.), Advances in experimental social psychology (Vol. 41, pp. 167–218). Academic Press. 10.1016/S0065-2601(08)00404-8 PMC2884406 20552040

[bibr9-17456916251414030] Baumgartner T. Speck D. Wettstein D. Masnari O. Beeli G. Jäncke L. (2008). Feeling present in arousing virtual reality worlds: Prefrontal brain regions differentially orchestrate presence experience in adults and children. Frontiers in Human Neuroscience, 2, Article 8. 10.3389/neuro.09.008.2008 PMC2572200 18958209

[bibr10-17456916251414030] Bennett P. R. McDonald K. B. (2013). Military service as a pathway to early socioeconomic achievement for disadvantaged groups. In Wilmoth J. M. London A. S. (Eds.), Life course perspectives on military service. Routledge.

[bibr11-17456916251414030] Berkman M. I. Akan E. (2019). Presence and immersion in virtual reality. In Lee N. (Ed.), Encyclopedia of computer graphics and games. Springer. 10.1007/978-3-319-08234-9_162-1

[bibr12-17456916251414030] Berthiaume M. Corno G. Nolet K. Bouchard S. (2018). A novel integrated information processing model of presence. Presence: Teleoperators and Virtual Environments, 27(4), 378–399. 10.1162/pres_a_00336

[bibr13-17456916251414030] Biocca F. A. Inoue Y. Lee A. Polinsky H. Tang A. (2002). Visual cues and virtual touch: Role of visual stimuli and intersensory integration in cross-modal haptic illusions and the sense of presence. In Gouveia F. (Ed.), Proceedings of presence 2002 (pp. 410–428). Fernando Pessoa University Press.

[bibr14-17456916251414030] Blankenburg W. (1971). Der Verlust der natürlichen Selbstverständlichkeit: Ein Beitrag zur Psychopathologie symptomarmer Schizophrenien [The loss of natural self-evidence: A contribution to the psychopathology of symptom-poor schizophrenia]. Enke.

[bibr15-17456916251414030] Bohil C. J. Alicea B. Biocca F. A. (2011). Virtual reality in neuroscience research and therapy. Nature Reviews Neuroscience, 12(12), 752–762. 10.1038/nrn3122 22048061

[bibr16-17456916251414030] Bormann K. (2005). Presence and the utility of audio spatialization. Presence: Teleoperators and Virtual Environments, 14(3), 278–297. 10.1162/105474605323384645

[bibr17-17456916251414030] Botella C. García-Palacios A. Villa H. Baños R. M. Quero S. Alcañiz M. Riva G. (2007). Virtual reality exposure in the treatment of panic disorder and agoraphobia: A controlled study. Clinical Psychology & Psychotherapy, 14(3), 164–175. 10.1002/cpp.524

[bibr18-17456916251414030] Brewin C. R. Gregory J. D. Lipton M. Burgess N. (2010). Intrusive images in psychological disorders: Characteristics, neural mechanisms, and treatment implications. Psychological Review, 117(1), 210–232. 10.1037/a0018113 20063969 PMC2834572

[bibr19-17456916251414030] Burdea G. Coiffet P. (2024). Virtual reality technology. John Wiley & Sons. 10.1002/9781119512608

[bibr20-17456916251414030] Camber R . (2024, January 1). Police launch the first investigation into ‘virtual rape.’ Mail Online. https://www.dailymail.co.uk/news/article-12917329/Police-launch-investigation-kind-virtual-rape-metaverse.html

[bibr21-17456916251414030] Carlin A. S. Hoffman H. G. Weghorst S. (1997). Virtual reality and tactile augmentation in the treatment of spider phobia: A case report. Behaviour Research and Therapy, 35(2), 153–158. 10.1016/s0005-7967(96)00085-x 9046678

[bibr22-17456916251414030] Clark A. (2013). Whatever next? Predictive brains, situated agents, and the future of cognitive science. Behavioral and Brain Sciences, 36(3), 181–204. 10.1017/S0140525X12000477 23663408

[bibr23-17456916251414030] Clark A. (2015). Radical predictive processing. The Southern Journal of Philosophy, 53(Suppl. 1), 3–27. 10.1111/sjp.12120

[bibr24-17456916251414030] Clemente M. Rey B. Rodríguez-Pujadas A. Barros-Loscertales A. Baños R. M. Botella C. Alcañiz M. Ávila C. (2014). An fMRI study to analyze neural correlates of presence during virtual reality experiences. Interacting with Computers, 26(3), 269–284. 10.1093/iwc/iwt037

[bibr25-17456916251414030] Clode D. Dowdall L. da Silva E. Selén K. Cowie D. Dominijanni G. Makin T. R. (2024). Evaluating initial usability of a hand augmentation device across a large and diverse sample. Science Robotics, 9(90), Article eadk5183. 10.1126/scirobotics.adk5183 PMC7616312 38809995

[bibr26-17456916251414030] Coelho C. Tichon J. Hine T. J. Wallis G. Riva G. (2006). Media presence and inner presence: The sense of presence in virtual reality technologies. In Riva G. Anguera M. T. Wiederhold B. K. Mantovani F. (Eds.), From communication to presence: Cognition, emotions and culture towards the ultimate communicative experience: Festschrift in honor of Luigi Anolli (pp. 25–45). IOS Press.

[bibr27-17456916251414030] Cooper N. Milella F. Pinto C. Cant I. White M. Meyer G. (2018). The effects of substitute multisensory feedback on task performance and the sense of presence in a virtual reality environment. PLOS One, 13(2), Article e0191846. 10.1371/journal.pone.0191846 PMC5794113 29390023

[bibr28-17456916251414030] Cornelio P. Velasco C. Obrist M. (2021). Multisensory integration as per technological advances: A review. Frontiers in Neuroscience, 15, Article 652611. 10.3389/fnins.2021.652611 PMC8257956 34239410

[bibr29-17456916251414030] Crucianelli L. Krahé C. Jenkinson P. M. Fotopoulou A. (2018). Interoceptive ingredients of body ownership: Affective touch and cardiac awareness in the rubber hand illusion. Cortex, 104, 180–192. 10.1016/j.cortex.2017.04.018 28532579

[bibr30-17456916251414030] Derntl B. Regenbogen C. (2014). Empathy. In Lysaker P. H. Dimaggio G. Brüne M. (Eds.), Social cognition and metacognition in schizophrenia (pp. 69–81). Academic Press. 10.1016/B978-0-12-405172-0.00004-1

[bibr31-17456916251414030] Deroy O. Spence C. Noppeney U. (2016). Metacognition in multisensory perception. Trends in Cognitive Sciences, 20(10), 736–747. 10.1016/j.tics.2016.08.006 27612983

[bibr32-17456916251414030] Deshmukh V. D. (2022). Consciousness, awareness, and presence: A neurobiological perspective. International Journal of Yoga, 15(2), 144–149. 10.4103/ijoy.ijoy_77_22 36329768 PMC9623886

[bibr33-17456916251414030] Difede J. Cukor J. (2007). Virtual reality exposure therapy for the treatment of posttraumatic stress disorder following September 11, 2001. The Journal of Clinical Psychiatry, 68(11), 1682–1689. 10.4088/JCP.v68n1102 18052556

[bibr34-17456916251414030] Difede J. Rothbaum B. O. Rizzo A. A. Wyka K. Spielman L. Reist C. Roy M. J. Jovanovic T. Norrholm S. D. Cukor J. Olden M. Glatt C. E. Lee F. S. (2022). Enhancing exposure therapy for posttraumatic stress disorder (PTSD): A randomized clinical trial of virtual reality and imaginal exposure with a cognitive enhancer. Translational Psychiatry, 12, Article 299. 10.1038/s41398-022-02066-x PMC9329292 35896533

[bibr35-17456916251414030] Dokic J. Martin J.-R. (2017). Felt reality and the opacity of perception. Topoi, 36(2), 299–309. 10.1007/s11245-015-9327-2

[bibr36-17456916251414030] Drori G. Bar-Tal P. Stern Y. Zvilichovsky Y. Salomon R. (2020). UnReal? Investigating the sense of reality and psychotic symptoms with virtual reality. Journal of Clinical Medicine, 9(6), 1627. 10.3390/jcm9061627 32481568 PMC7355917

[bibr37-17456916251414030] Dürschmid S. Edwards E. Reichert C. Dewar C. Hinrichs H. Heinze H.-J. Kirsch H. E. Dalal S. S. Deouell L. Y. Knight R. T. (2016). Hierarchy of prediction errors for auditory events in human temporal and frontal cortex. Proceedings of the National Academy of Sciences, USA, 113(24), 6755–6760. 10.1073/pnas.1525030113 PMC4914143 27247381

[bibr38-17456916251414030] Ehrsson H. H. (2012). The concept of body ownership and its relation to multisensory integration. In Stein B. E. (Ed.), The new handbook of multisensory processes (pp. 775–792). MIT Press. 10.7551/mitpress/8466.003.0067

[bibr39-17456916251414030] Eshuis L. V. van Gelderen M. J. van Zuiden M. Nijdam M. J. Vermetten E. Olff M. Bakker A. (2021). Efficacy of immersive PTSD treatments: A systematic review of virtual and augmented reality exposure therapy and a meta-analysis of virtual reality exposure therapy. Journal of Psychiatric Research, 143, 516–527. 10.1016/j.jpsychires.2020.11.030 33248674

[bibr40-17456916251414030] Felnhofer A. Pfannerstill F. Gänsler L. Kothgassner O. D. Humer E. Büttner J. Probst T. (2025). Barriers to adopting therapeutic virtual reality: The perspective of clinical psychologists and psychotherapists. Frontiers in Psychiatry, 16, Article 1549090. 10.3389/fpsyt.2025.1549090 PMC11958971 40171310

[bibr41-17456916251414030] Felton W. M. Jackson R. E. (2022). Presence: A review. International Journal of Human–Computer Interaction, 38(1), 1–18. 10.1080/10447318.2021.1921368

[bibr42-17456916251414030] Fernández Velasco P. Loev S . (2024). Metacognitive feelings: A predictive-processing perspective. Perspectives on Psychological Science, 20(4), 691–713. 10.1177/17456916231221976 38285929 PMC12231856

[bibr43-17456916251414030] Filippetti M. L. Kirsch L. P. Crucianelli L. Fotopoulou A. (2019). Affective certainty and congruency of touch modulate the experience of the rubber hand illusion. Scientific Reports, 9, Article 2635. 10.1038/s41598-019-38880-5 PMC6385173 30796333

[bibr44-17456916251414030] Fortier M. (2018). Sense of reality, metacognition, and culture in schizophrenic and drug-induced hallucinations: An interdisciplinary approach. In Proust J. Fortier M. (Eds.), Metacognitive diversity: An interdisciplinary approach (pp. 343–378). Oxford University Press. 10.1093/oso/9780198789710.003.0016

[bibr45-17456916251414030] Fotopoulou A. Jenkinson P. M. Tsakiris M. Haggard P. Rudd A. Kopelman M. D. (2011). Mirror-view reverses somatoparaphrenia: Dissociation between first- and third-person perspectives on body ownership. Neuropsychologia, 49(14), 3946–3955. 10.1016/j.neuropsychologia.2011.10.011 22023911

[bibr46-17456916251414030] Friston K. Kilner J. Harrison L. (2006). A free energy principle for the brain. Journal of Physiology-Paris, 100(1), 70–87. 10.1016/j.jphysparis.2006.10.001 17097864

[bibr47-17456916251414030] Friston K. Rigoli F. Ognibene D. Mathys C. Fitzgerald T. Pezzulo G. (2015). Active inference and epistemic value. Cognitive Neuroscience, 6(4), 187–214. 10.1080/17588928.2015.1020053 25689102

[bibr48-17456916251414030] Gallace A. Ngo M. K. Sulaitis J. Spence C. (2012). Multisensory presence in virtual reality: Possibilities and limitations. In Ghinea G. Andres F. Gulliver S. (Eds.), Multiple sensorial media advances and applications: New developments in MulSeMedia (pp. 1–38). IGI Global Scientific Publishing. 10.4018/978-1-60960-821-7.ch001

[bibr49-17456916251414030] Garcia-Palacios A. Hoffman H. Carlin A. Furness T. A. Botella C. (2002). Virtual reality in the treatment of spider phobia: A controlled study. Behaviour Research and Therapy, 40(9), 983–993. 10.1016/S0005-7967(01)00068-7 12296495

[bibr50-17456916251414030] Garfinkel S. N. Nagai Y. Seth A. K. Critchley H. D. (2013). Neuroimaging studies of interoception and self-awareness. In Cavanna A. E. Nani A. Blumenfeld H. Laureys S. (Eds.), Neuroimaging of consciousness (pp. 207–224). Springer. 10.1007/978-3-642-37580-4_11

[bibr51-17456916251414030] Gershuny B. S. Thayer J. F. (1999). Relations among psychological trauma, dissociative phenomena, and trauma-related distress: A review and integration. Clinical Psychology Review, 19(5), 631–657. 10.1016/s0272-7358(98)00103-2 10467494

[bibr52-17456916251414030] Gibbs J. K. Gillies M. Pan X. (2022). A comparison of the effects of haptic and visual feedback on presence in virtual reality. International Journal of Human-Computer Studies, 157, 102717. 10.1016/j.ijhcs.2021.102717

[bibr53-17456916251414030] Gonçalves R. Pedrozo A. L. Coutinho E. S. F. Figueira I. Ventura P. (2012). Efficacy of virtual reality exposure therapy in the treatment of PTSD: A systematic review. PLOS One, 7(12), Article e48469. 10.1371/journal.pone.0048469 PMC3531396 23300515

[bibr54-17456916251414030] Grassini S. Laumann K. (2020). Questionnaire measures and physiological correlates of presence: A systematic review. Frontiers in Psychology, 11, Article 349. https://www.frontiersin.org/articles/10.3389/fpsyg.2020.00349 10.3389/fpsyg.2020.00349 PMC7096541 32265769

[bibr55-17456916251414030] Grassini S. Laumann K. Thorp S. Topranin V. D. M. (2021). Using electrophysiological measures to evaluate the sense of presence in immersive virtual environments: An event-related potential study. Brain and Behavior, 11(8), Article e2269. 10.1002/brb3.2269 PMC8413821 34173347

[bibr56-17456916251414030] Heilbron M. Chait M. (2018). Great expectations: Is there evidence for predictive coding in auditory cortex? Neuroscience, 389, 54–73. 10.1016/j.neuroscience.2017.07.061 28782642

[bibr57-17456916251414030] Iachini T. Maffei L. Masullo M. Senese V. P. Rapuano M. Pascale A. Sorrentino F. Ruggiero G. (2019). The experience of virtual reality: Are individual differences in mental imagery associated with sense of presence? Cognitive Processing, 20(3), 291–298. 10.1007/s10339-018-0897-y 30569268

[bibr58-17456916251414030] International Society for Telepresence Research. (2025). Presence defined. https://ispr.info/about-presence-2/about-presence

[bibr59-17456916251414030] Jaspers K. (1913). General psychopathology. Johns Hopkins University Press.

[bibr60-17456916251414030] Jenkinson P. M. Moro V. Fotopoulou A. (2022). Disorders of body ownership. In Alsmith A. Longo M. (Eds.), The Routledge handbook of bodily awareness. Routledge. 10.4324/9780429321542-34

[bibr61-17456916251414030] Jicol C. Clarke C. Tor E. Yip H. L. Yoon J. Bevan C. Bowden H. Brann E. Cater K. Cole R. Deeley Q. Eidinow E. O’Neill E. Lutteroth C. Proulx M. J. (2023). Imagine that! Imaginative suggestibility affects presence in virtual reality. In Schmidt A. , et al. (Eds.), CHI '23: Proceedings of the 2023 CHI Conference on Human Factors in Computing Systems (pp. 1–11). 10.1145/3544548.3581212

[bibr62-17456916251414030] Johnsdorf M. Kisker J. Gruber T. Schöne B. (2023). Comparing encoding mechanisms in realistic virtual reality and conventional 2D laboratory settings: Event-related potentials in a repetition suppression paradigm. Frontiers in Psychology, 14, Article 1051938. 10.3389/fpsyg.2023.1051938 PMC9912617 36777234

[bibr63-17456916251414030] Johnson M. K. Raye C. L. (1981). Reality monitoring. Psychological Review, 88(1), 67–85. 10.1037/0033-295X.88.1.67

[bibr64-17456916251414030] Jones S. Dawkins S. (2018). The Sensorama revisited: Evaluating the application of multi-sensory input on the sense of presence in 360-degree immersive film in virtual reality. In Jung T. tom Dieck M. C. (Eds.), Augmented reality and virtual reality: Empowering human, place and business (pp. 183–197). Springer. 10.1007/978-3-319-64027-3_13

[bibr65-17456916251414030] Karpinski A. C. Scullin M. H. (2009). Suggestibility under pressure: Theory of mind, executive function, and suggestibility in preschoolers. Journal of Applied Developmental Psychology, 30(6), 749–763. 10.1016/j.appdev.2009.05.004

[bibr66-17456916251414030] Khalsa S. S. Adolphs R. Cameron O. G. Critchley H. D. Davenport P. W. Feinstein J. S. Feusner J. D. Garfinkel S. N. Lane R. D. Mehling W. E. Meuret A. E. Nemeroff C. B. Oppenheimer S. Petzschner F. H. Pollatos O. Rhudy J. L. Schramm L. P. Simmons W. K. Stein M. B. . . . Zucker N. (2018). Interoception and mental health: A roadmap. Biological Psychiatry: Cognitive Neuroscience and Neuroimaging, 3(6), 501–513. 10.1016/j.bpsc.2017.12.004 29884281 PMC6054486

[bibr67-17456916251414030] Kim H. Lee I.-K. (2022). Studying the effects of congruence of auditory and visual stimuli on virtual reality experiences. IEEE Transactions on Visualization and Computer Graphics, 28(5), 2080–2090. 10.1109/TVCG.2022.3150514 35167477

[bibr68-17456916251414030] Kisker J. Johnsdorf M. Sagehorn M. Schöne B. Gruber T. (2024). Induced oscillatory brain responses under virtual reality conditions in the context of repetition priming. Experimental Brain Research, 242(3), 525–541. 10.1007/s00221-023-06766-8 38200371 PMC10894769

[bibr69-17456916251414030] Klinger E. Bouchard S. Légeron P. Roy S. Lauer F. Chemin I. Nugues P. (2005). Virtual reality therapy versus cognitive behavior therapy for social phobia: A preliminary controlled study. CyberPsychology & Behavior, 8(1), 76–88. 10.1089/cpb.2005.8.76 15738695

[bibr70-17456916251414030] Kober S. E. Kurzmann J. Neuper C. (2012). Cortical correlate of spatial presence in 2D and 3D interactive virtual reality: An EEG study. International Journal of Psychophysiology, 83(3), 365–374. 10.1016/j.ijpsycho.2011.12.003 22206906

[bibr71-17456916251414030] Kothgassner O. D. Goreis A. Kafka J. X. Van Eickels R. L. Plener P. L. Felnhofer A. (2019). Virtual reality exposure therapy for posttraumatic stress disorder (PTSD): A meta-analysis. European Journal of Psychotraumatology, 10(1), Article 1654782. 10.1080/20008198.2019.1654782 PMC6713125 31489138

[bibr72-17456916251414030] Kreimeier J. Hammer S. Friedmann D. Karg P. Bühner C. Bankel L. Götzelmann T. (2019). Evaluation of different types of haptic feedback influencing the task-based presence and performance in virtual reality. In Proceedings of the 12th ACM International Conference on PErvasive Technologies Related to Assistive Environments (pp. 289–298). Association for Computing Machinery. 10.1145/3316782.3321536

[bibr73-17456916251414030] Krijn M. Emmelkamp P. M. G. Biemond R. de Wilde de Ligny C. Schuemie M. J. van der Mast C. A. P. G . (2004). Treatment of acrophobia in virtual reality: The role of immersion and presence. Behaviour Research and Therapy, 42(2), 229–239. 10.1016/S0005-7967(03)00139-6 14975783

[bibr74-17456916251414030] Lavoie R. Main K. King C. King D. (2021). Virtual experience, real consequences: The potential negative emotional consequences of virtual reality gameplay. Virtual Reality, 25(1), 69–81. 10.1007/s10055-020-00440-y

[bibr75-17456916251414030] Lemche E. Sierra-Siegert M. David A. S. Phillips M. L. Gasston D. Williams S. C. R. Giampietro V. P. (2016). Cognitive load and autonomic response patterns under negative priming demand in depersonalization-derealization disorder. The European Journal of Neuroscience, 43(7), 971–978. 10.1111/ejn.13183 26791018 PMC4855951

[bibr76-17456916251414030] Lessiter J. Freeman J. Keogh E. Davidoff J. (2001). A cross-media presence questionnaire: The ITC-Sense of Presence Inventory. Presence: Teleoperators and Virtual Environments, 10(3), 282–297. 10.1162/105474601300343612

[bibr77-17456916251414030] Lin T. J.-H. (2017). Fear in virtual reality (VR): Fear elements, coping reactions, immediate and next-day fright responses toward a survival horror zombie virtual reality game. Computers in Human Behavior, 72, 350–361. 10.1016/j.chb.2017.02.057

[bibr78-17456916251414030] Lin T. J.-H. (2023). Virtual reality horror games and fear in gaming. In Powers M. (Ed.), Oxford encyclopedia of communication. Oxford University Press. 10.1093/acrefore/9780190228613.013.1478

[bibr79-17456916251414030] López-Silva P. (2018). Mapping the psychotic mind: A review on the subjective structure of thought insertion. Psychiatric Quarterly, 89(4), 957–968. 10.1007/s11126-018-9593-4 30090993

[bibr80-17456916251414030] Marucci M. Di Flumeri G. Borghini G. Sciaraffa N. Scandola M. Pavone E. F. Babiloni F. Betti V. Aricò P. (2021). The impact of multisensory integration and perceptual load in virtual reality settings on performance, workload and presence. Scientific Reports, 11, Article 4831. 10.1038/s41598-021-84196-8 PMC7921449 33649348

[bibr81-17456916251414030] Medford N. Sierra M. Stringaris A. Giampietro V. Brammer M. J. David A. S. (2016). Emotional experience and awareness of self: Functional MRI studies of depersonalization disorder. Frontiers in Psychology, 7, Article 432. 10.3389/fpsyg.2016.00432 PMC4890597 27313548

[bibr82-17456916251414030] Meehan M. Insko B. Whitton M. Brooks F. P. (2002). Physiological measures of presence in stressful virtual environments. ACM Transactions on Graphics, 21(3), 645–652. 10.1145/566654.566630

[bibr83-17456916251414030] Metzinger T. (2003). Phenomenal transparency and cognitive self-reference. Phenomenology and the Cognitive Sciences, 2(4), 353–393. 10.1023/B:PHEN.0000007366.42918.eb

[bibr84-17456916251414030] Metzinger T. (2014). How does the brain encode epistemic reliability? Perceptual presence, phenomenal transparency, and counterfactual richness. Cognitive Neuroscience, 5(2), 122–124. 10.1080/17588928.2014.905519 24702471

[bibr85-17456916251414030] Mühlberger A. Bülthoff H. H. Wiedemann G. Pauli P. (2007). Virtual reality for the psychophysiological assessment of phobic fear: Responses during virtual tunnel driving. Psychological Assessment, 19(3), 340–346. 10.1037/1040-3590.19.3.340 17845125

[bibr86-17456916251414030] Murphy R. J. (2023). Depersonalization/derealization disorder and neural correlates of trauma-related pathology: A critical review. Innovations in Clinical Neuroscience, 20(1–3), 53–59. PMC10132272 37122581

[bibr87-17456916251414030] Nisbett R. E. Peng K. Choi I. Norenzayan A. (2001). Culture and systems of thought: Holistic versus analytic cognition. Psychological Review, 108(2), 291–310. 10.1037/0033-295X.108.2.291 11381831

[bibr88-17456916251414030] Norenzayan A. Smith E. E. Kim B. J. Nisbett R. E. (2002). Cultural preferences for formal versus intuitive reasoning. Cognitive Science, 26(5), 653–684. 10.1207/s15516709cog2605_4

[bibr89-17456916251414030] Norr A. M. Smolenski D. J. Reger G. M. (2018). Virtual reality exposure versus prolonged exposure for PTSD: Which treatment for whom? Depression and Anxiety, 35(6), 523–529. 10.1002/da.22751 29734488

[bibr90-17456916251414030] Nunez D. Blake E. (2001). Cognitive presence as a unified concept of virtual reality effectiveness. In AFRIGRAPH '01: Proceedings of the 1st International Conference on Computer Graphics, Virtual Reality and Visualisation (pp. 115–118). Association for Computing Machinery. 10.1145/513867.513892

[bibr91-17456916251414030] Ogawa A. Bordier C. Macaluso E. (2013). Amygdala activation is associated with sense of presence during viewing 3D-surround cinematography. In Lee M. Hirose A. Hou Z.-G. Kil R. M. (Eds.), Neural information processing (pp. 153–160). Springer. 10.1007/978-3-642-42054-2_20

[bibr92-17456916251414030] Palmiero M. Piccardi L. Giancola M. Nori R. D’Amico S. Olivetti Belardinelli M. (2019). The format of mental imagery: From a critical review to an integrated embodied representation approach. Cognitive Processing, 20(3), 277–289. 10.1007/s10339-019-00908-z 30798484

[bibr93-17456916251414030] Peter M. G. Porada D. K. Regenbogen C. Olsson M. J. Lundström J. N. (2019). Sensory loss enhances multisensory integration performance. Cortex, 120, 116–130. 10.1016/j.cortex.2019.06.003 31299497

[bibr94-17456916251414030] Phillips M. L. Medford N. Senior C. Bullmore E. T. Suckling J. Brammer M. J. Andrew C. Sierra M. Williams S. C. R. David A. S. (2001). Depersonalization disorder: Thinking without feeling. Psychiatry Research: Neuroimaging, 108(3), 145–160. 10.1016/S0925-4927(01)00119-6 11756013

[bibr95-17456916251414030] Pianzola F. Riva G. Kukkonen K. Mantovani F. (2021). Presence, flow, and narrative absorption: An interdisciplinary theoretical exploration with a new spatiotemporal integrated model based on predictive processing. Open Research Europe. 10.12688/openreseurope.13193.2 PMC10446082 37645177

[bibr96-17456916251414030] Porta C. M. Frerich E. A. Hoffman S. Bauer S. Jain V. M. Bradley C. (2024). Sexual violence in virtual reality: A scoping review. Journal of Forensic Nursing, 20(1), 66–77. 10.1097/JFN.0000000000000466 38093420 PMC11841731

[bibr97-17456916251414030] Powers M. B. Emmelkamp P. M. G. (2008). Virtual reality exposure therapy for anxiety disorders: A meta-analysis. Journal of Anxiety Disorders, 22(3), 561–569. 10.1016/j.janxdis.2007.04.006 17544252

[bibr98-17456916251414030] Preiss D. D. (2022). Metacognition, mind wandering, and cognitive flexibility: Understanding creativity. Journal of Intelligence, 10(3), 69. 10.3390/jintelligence10030069 36135610 PMC9500634

[bibr99-17456916251414030] Rabellino D. Burin D. Harricharan S. Lloyd C. Frewen P. A. McKinnon M. C. Lanius R. A. (2018). Altered sense of body ownership and agency in posttraumatic stress disorder and its dissociative subtype: A rubber hand illusion study. Frontiers in Human Neuroscience, 12, Article 163. 10.3389/fnhum.2018.00163 PMC5938392 29765311

[bibr100-17456916251414030] Ratcliffe M. (2008). Feelings of being: Phenomenology, psychiatry and the sense of reality. Oxford University Press.

[bibr101-17456916251414030] Reger G. M. Koenen-Woods P. Zetocha K. Smolenski D. J. Holloway K. M. Rothbaum B. O. Difede J. Rizzo A. A. Edwards-Stewart A. Skopp N. A. Mishkind M. Reger M. A. Gahm G. A. (2016). Randomized controlled trial of prolonged exposure using imaginal exposure vs. virtual reality exposure in active duty soldiers with deployment-related posttraumatic stress disorder (PTSD). Journal of Consulting and Clinical Psychology, 84(11), 946–959. 10.1037/ccp0000134 27606699

[bibr102-17456916251414030] Revonsuo A. (2006). Inner presence: Consciousness as a biological phenomenon. MIT Press.

[bibr103-17456916251414030] Richardson L. K. Frueh B. C. Acierno R. (2010). Prevalence estimates of combat-related PTSD: A critical review. The Australian and New Zealand Journal of Psychiatry, 44(1), 4–19. 10.3109/00048670903393597 20073563 PMC2891773

[bibr104-17456916251414030] Riva G. Mantovani F. (2012). From the body to the tools and back: A general framework for presence in mediated interactions. Interacting with Computers, 24(4), 203–210. 10.1016/j.intcom.2012.04.007

[bibr105-17456916251414030] Rothbaum B. O. Anderson P. Zimand E. Hodges L. Lang D. Wilson J. (2006). Virtual reality exposure therapy and standard (in vivo) exposure therapy in the treatment of fear of flying. Behavior Therapy, 37(1), 80–90. 10.1016/j.beth.2005.04.004 16942963

[bibr106-17456916251414030] Rumsey F. (2021). Are you there? Presence and practice in immersive audio. Audio Engineering Society Convention Papers Forum. https://secure.aes.org/forum/pubs/conventions/?elib=20926

[bibr107-17456916251414030] Rus-Calafell M. Garety P. Sason E. Craig T. J. K. Valmaggia L. R. (2018). Virtual reality in the assessment and treatment of psychosis: A systematic review of its utility, acceptability and effectiveness. Psychological Medicine, 48(3), 362–391. 10.1017/S0033291717001945 28735593

[bibr108-17456916251414030] Safikhani S. Gattringer V. Schmied M. Pirker J. Wriessnegger S. C. (2024). The influence of realism on the sense of presence in virtual reality: Neurophysiological insights using EEG. Multimodal Technologies and Interaction, 8(11), Article 11. 10.3390/mti8110104

[bibr109-17456916251414030] Sales N. J. (2024, January 5). A girl was allegedly raped in the metaverse. Is this the beginning of a dark new future? The Guardian. https://www.theguardian.com/commentisfree/2024/jan/05/metaverse-sexual-assault-vr-game-online-safety-meta

[bibr110-17456916251414030] Sanchez-Vives M. V. Slater M. (2005). From presence to consciousness through virtual reality. Nature Reviews Neuroscience, 6(4), 332–339. 10.1038/nrn1651 15803164

[bibr111-17456916251414030] Sas C. O’Hare G. M. P. (2003). Presence equation: An investigation into cognitive factors underlying presence. Presence: Teleoperators and Virtual Environments, 12(5), 523–537. 10.1162/105474603322761315

[bibr112-17456916251414030] Šašinková A. Cˇeneˇk J. Ugwitz P. Tsai J.-L. Giannopoulos I. Lacko D. Stachonˇ Z. Fitz J. Šašinka Cˇ . (2023). Exploring cross-cultural variations in visual attention patterns inside and outside national borders using immersive virtual reality. Scientific Reports, 13, Article 18852. 10.1038/s41598-023-46103-1 PMC10620163 37914809

[bibr113-17456916251414030] Schwengerer L. (2019). Self-knowledge in a predictive processing framework. Review of Philosophy and Psychology, 10(3), 563–585. 10.1007/s13164-018-0416-1

[bibr114-17456916251414030] Senzaki S. Masuda T. Ishii K. (2014). When is perception top-down and when is it not? Culture, narrative, and attention. Cognitive Science, 38(7), 1493–1506. 10.1111/cogs.12118 24646291

[bibr115-17456916251414030] Seth A. K. (2014). A predictive processing theory of sensorimotor contingencies: Explaining the puzzle of perceptual presence and its absence in synesthesia. Cognitive Neuroscience, 5(2), 97–118. 10.1080/17588928.2013.877880 24446823 PMC4037840

[bibr116-17456916251414030] Seth A. K. Suzuki K. Critchley H. D. (2012). An interoceptive predictive coding model of conscious presence. Frontiers in Psychology, 2, Article 395. 10.3389/fpsyg.2011.00395 PMC3254200 22291673

[bibr117-17456916251414030] Slater M. (2018). Immersion and the illusion of presence in virtual reality. British Journal of Psychology, 109(3), 431–433. 10.1111/bjop.12305 29781508

[bibr118-17456916251414030] Slater M. Spanlang B. Sanchez-Vives M. V. Blanke O. (2010). First person experience of body transfer in virtual reality. PLOS One, 5(5), Article e10564. 10.1371/journal.pone.0010564 PMC2868878 20485681

[bibr119-17456916251414030] Steuer J. (1992). Defining virtual reality: Dimensions determining telepresence. Journal of Communication, 42(4), 73–93. 10.1111/j.1460-2466.1992.tb00812.x

[bibr120-17456916251414030] Suzuki K. Garfinkel S. N. Critchley H. D. Seth A. K. (2013). Multisensory integration across exteroceptive and interoceptive domains modulates self-experience in the rubber-hand illusion. Neuropsychologia, 51(13), 2909–2917. 10.1016/j.neuropsychologia.2013.08.014 23993906

[bibr121-17456916251414030] Suzuki K. Mariola A. Schwartzman D. J. Seth A. K. (2023). Using extended reality to study the experience of presence. In Maymon C. Grimshaw G. Wu Y. C. (Eds.), Virtual reality in behavioral neuroscience: New insights and methods (pp. 255–285). Springer. 10.1007/7854_2022_401 36592275

[bibr122-17456916251414030] Tang M. F. Smout C. A. Arabzadeh E. Mattingley J. B. (2018). Prediction error and repetition suppression have distinct effects on neural representations of visual information. eLife, 7, Article e33123. 10.7554/eLife.33123 PMC6312401 30547881

[bibr123-17456916251414030] Tateo L. (2016). What imagination can teach us about higher mental functions. In Valsiner J. Marsico G. Chaudhary N. Sato T. Dazzani V. (Eds.), Psychology as the science of human being: The Yokohama manifesto (pp. 149–164). Springer. 10.1007/978-3-319-21094-0_9

[bibr124-17456916251414030] Tortella-Feliu M. Botella C. Llabrés J. Bretón-López J. M. del Amo A. R. Baños R. M. Gelabert J. M. (2011). Virtual reality versus computer-aided exposure treatments for fear of flying. Behavior Modification, 35(1), 3–30. 10.1177/0145445510390801 21177516

[bibr125-17456916251414030] Toussaint B. Heinzle J. Stephan K. E. (2024). A computationally informed distinction of interoception and exteroception. Neuroscience & Biobehavioral Reviews, 159, Article 105608. 10.1016/j.neubiorev.2024.105608 38432449

[bibr126-17456916251414030] Tran T. Q. Langlotz T. Young J. Schubert T. W. Regenbrecht H. (2024). Classifying presence scores: Insights and analysis from two decades of the Igroup Presence Questionnaire (IPQ). ACM Transactions on Computer-Human Interaction, 31(5), Article 61. 10.1145/3689046

[bibr127-17456916251414030] Triberti S. Brivio E. Galimberti C. (2018). On social presence: Theories, methodologies, and guidelines for the innovative contexts of computer-mediated learning. In Marmon M. (Ed.), Enhancing social presence in online learning environments (pp. 20–41). IGI Global Scientific Publishing. 10.4018/978-1-5225-3229-3.ch002

[bibr128-17456916251414030] Triberti S. Sapone C. Riva G. (2025). Being there but where? Sense of presence theory for virtual reality applications. Humanities and Social Sciences Communications, 12, Article 79. 10.1057/s41599-025-04380-3

[bibr129-17456916251414030] Usoh M. Catena E. Arman S. Slater M. (2000). Using presence questionnaires in reality. Presence: Teleoperators and Virtual Environments, 9(5), 497–503. 10.1162/105474600566989

[bibr130-17456916251414030] Valzolgher C. Mazzurega M. Zampini M. Pavani F. (2018). Incongruent multisensory stimuli alter bodily self-consciousness: Evidence from a first-person perspective experience. Acta Psychologica, 191, 261–270. 10.1016/j.actpsy.2018.09.009 30352360

[bibr131-17456916251414030] van Heugten-van der Kloet D. Cosgrave J. van Rheede J. Hicks S . (2018). Out-of-body experience in virtual reality induces acute dissociation. Psychology of Consciousness: Theory, Research, and Practice, 5(4), 346–357. 10.1037/cns0000172

[bibr132-17456916251414030] van Ulzen N. R. Semin G. R. Oudejans R. R. D. Beek P. J . (2008). Affective stimulus properties influence size perception and the Ebbinghaus illusion. Psychological Research, 72(3), 304–310. 10.1007/s00426-007-0114-6 17410379 PMC2668624

[bibr133-17456916251414030] Wacongne C. Labyt E. van Wassenhove V. Bekinschtein T. Naccache L. Dehaene S. (2011). Evidence for a hierarchy of predictions and prediction errors in human cortex. Proceedings of the National Academy of Sciences, USA, 108(51), 20754–20759. 10.1073/pnas.1117807108 PMC3251061 22147913

[bibr134-17456916251414030] Walsh K. S. McGovern D. P. Clark A. O’Connell R. G. (2020). Evaluating the neurophysiological evidence for predictive processing as a model of perception. Annals of the New York Academy of Sciences, 1464(1), 242–268. 10.1111/nyas.14321 32147856 PMC7187369

[bibr135-17456916251414030] Waterworth J. A. Waterworth E. L. Mantovani F. Riva G. (2010). On feeling (the) present: An evolutionary account of the sense of presence in physical and electronically-mediated environments. Journal of Consciousness Studies, 17(1–2), 167–188.

[bibr136-17456916251414030] Witmer B. G. Singer M. J. (1998). Measuring presence in virtual environments: A presence questionnaire. Presence: Teleoperators and Virtual Environments, 7(3), 225–240. 10.1162/105474698565686

[bibr137-17456916251414030] Wormwood J. B. Siegel E. H. Kopec J. Quigley K. S. Barrett L. F. (2019). You are what I feel: A test of the affective realism hypothesis. Emotion, 19(5), 788–798. 10.1037/emo0000484 30138005

[bibr138-17456916251414030] Yang J. Millman L. S. M. David A. S. Hunter E. C. M. (2023). The prevalence of depersonalization-derealization disorder: A systematic review. Journal of Trauma & Dissociation, 24(1), 8–41. 10.1080/15299732.2022.2079796 35699456

